# Opioid Peptide Hybrids as Multifunctional Analgesic Ligands: Emphasis on Intracellular Signaling

**DOI:** 10.3390/ph19101550

**Published:** 2026-09-30

**Authors:** Igor Cecherz, Jakub Waśkiewicz, Alicja Dudek, Dariusz Polatyński, Karol Wtorek, Justyna Piekielna-Ciesielska

**Affiliations:** Department of Biomolecular Chemistry, Medical University of Lodz, ul. Mazowiecka 6/8, 92-215 Lodz, Poland; igor.cecherz@student.umed.lodz.pl (I.C.); jakub.waskiewicz@student.umed.lodz.pl (J.W.); alicja.dudek@student.umed.lodz.pl (A.D.); dariusz.polatynski@student.umed.lodz.pl (D.P.); karol.wtorek@umed.lodz.pl (K.W.)

**Keywords:** opioid peptide hybrids, multifunctional analgesics, G protein, β-arrestin, MAPK, nociceptin/orphanin FQ peptide, neurokinin, neurotensin, NPFF, cholecystokinin, melanocortin receptors

## Abstract

Opioid peptide hybrids are multifunctional molecules in which an opioid pharmacophore is covalently integrated with a second opioid or non-opioid pharmacophore within a single molecular entity. Their development is driven by a major challenge in analgesic pharmacotherapy: although μ-opioid receptor (MOR) agonists remain among the most effective agents for the treatment of moderate-to-severe pain, their therapeutic use is limited by adverse effects and risks associated particularly with prolonged or high-dose exposure, including tolerance, constipation, respiratory depression, physical dependence, and abuse liability. Especially, chronic and neuropathic pain involves complex interactions among multiple receptor systems, neurotransmitter networks, and intracellular signaling pathways. Accordingly, hybrid peptide ligands provide a rational multitarget strategy aimed at achieving analgesia through the coordinated modulation of complementary pharmacological targets rather than selective modulation of a single receptor system. This approach may enhance analgesic efficacy and potentially improve the therapeutic profile of opioid-based treatments by reducing some of the dose-limiting adverse effects associated with conventional opioid analgesics. In this review, we discuss the functional and pharmacological interactions between opioid receptors and other receptor systems involved in pain perception and modulation, with emphasis on those for which peptide-based hybrid ligands with demonstrated antinociceptive activity have been developed.

## 1. Introduction

Opioid receptors are the primary targets for the most potent antinociceptive drugs available in medicine. They belong to the massive family of G protein-coupled receptors (GPCRs) and are distributed extensively throughout the central nervous system (particularly in the brainstem and spinal cord) and in peripheral tissues. There are three main types of opioid receptor: μ-, δ-, and κ-opioid receptors, MOR, DOR, and KOR, respectively. Activation of the MOR is responsible for strongest antinociceptive effect; however, its prolonged stimulation leads to receptor desensitization, development of tolerance, dependence, respiratory depression, constipation, opioid-induced hyperalgesia (OIH), and withdrawal due to compensatory cellular adaptations. Classical opioids such as morphine and fentanyl are strong MOR agonists and remain the most effective analgesics available, yet their clinical use is hampered by those serious side effects [1,2,3].

A key pharmacological insight from the past three decades indicates that these adverse effects may be dissociated from analgesia, as they stem from specific intracellular signaling consequences triggered by activation of the MOR (β-arrestin 2 recruitment, adenylyl cyclase superactivation upon chronic treatment, ERK/MAPK signaling, receptor internalization, and desensitization) and from cross-talk with other systems involved in pain perception including nociceptin/orphanin FQ (N/OFQ, NOP) receptor, substance P/neurokinin-1 (NK1), neurotensin, cholecystokinin (CCK), melanocortin and neuropeptide FF (NPFF). Opioid hybrids are specific types of compounds which fuse an opioid pharmacophore with a second pharmacophore engaging another opioid subtype, or a non-opioid modulatory receptor.

Therefore, the concept behind the design of opioid hybrids involves simultaneously targeting two receptors whose signaling pathways converge to produce stronger analgesic effect but diverge regarding the pathways responsible for adverse effects [4,5,6,7]. The term “hybrid” is used in several related ways. A bifunctional ligand contains two pharmacophores in one molecule and is intended to interact with two pharmacological targets. A bivalent ligand contains two recognition elements connected by a spacer and may bridge receptor protomers or receptor heteromers. A multifunctional compound may activate or block more than one receptor without necessarily being long enough to physically bridge receptors. A merged pharmacophor contains overlapping structural elements that contribute to activity at both targets. Therefore, hybrid ligands may simply co-activate two independent receptors, bias one receptor toward a signaling pathway, stabilize receptor heteromers, or alter receptor trafficking [8,9].

From a signaling perspective, opioid hybrids should not be understood simply as “stronger opioids.” Their biological profile depends on how the opioid component engages G_i/o_ proteins, β-arrestins, receptor phosphorylation, desensitization, internalization, ERK/MAPK, PI3K/AKT and ion channel regulation, and on how the second pharmacophore modifies pronociceptive or anti-opioid systems.

Here we focus on opioid hybrid compounds of peptide structure. Peptides are attractive models for drug design because their scaffolds offer a structural solution to the biggest problem in opioid pharmacology: separating potent pain relief from dangerous side effects. Unlike traditional small-molecule opioids (like morphine or fentanyl), which are rigid and tend to bind deeply within the pocket of a single receptor type, peptide hybrids are modular. Therefore, the peptide structures may be designed to simultaneously target different specific interactions across multiple biological systems [10,11].

The opioid peptide pharmacophore can be derived from endogenous ligands, like enkephalins, endomorphins, dermorphin, deltorphin, dynorphin-like motifs, biphalin-like structures, or synthetic opioid peptides with unnatural or D-amino acids incorporated in their structure. A second pharmacophore can then be attached through a flexible, rigid, or overlapping linker to modulate another receptor system involved in pain perception. This strategy aims to combine analgesic mechanisms, reduce effective opioid dose, block anti-opioid pronociceptive pathways, alter receptor trafficking, or create receptor-specific signaling profiles [6,9,12,13,14,15,16].

Understanding the intracellular mechanisms responsible for therapeutic effect and the side effects of molecules which influence pain perception is crucial for detailing these specific metrics and providing the “blueprint” for drug design. This review provides the rational framework to translate chemical structure into physiological response, with a focus on current knowledge about the intracellular signaling of these molecules.

## 2. Opioid Receptor Signaling

Opioid receptors (MOR, DOR, KOR, and the nociceptin/orphanin FQ receptor) are class A of the G protein-coupled receptor family. Their activation produces analgesia mainly through G_i/o_-mediated inhibition of adenylyl cyclase, reduction of cAMP signaling, modulation of Ca^2+^ and K^+^ channels, reduced neurotransmitter release, and downstream regulation of kinases such as ERK/MAPK. In parallel, activated opioid receptors can be phosphorylated by GPCR kinases and recruit β-arrestins, which contribute to desensitization, internalization, recycling and additional signaling outputs [13,17].

The canonical opioid signaling pathway begins when an agonist stabilizes an active receptor conformation that couples to G_i/o_ proteins. The G_αi/o_ subunit inhibits adenylyl cyclase and lowers cAMP, while G_βγ_ subunits regulate ion channels, including inhibition of voltage-gated Ca^2+^ channels and activation of G protein-coupled inwardly rectifying K^+^ channels. These actions decrease neuronal excitability and neurotransmitter release in nociceptive pathways [17,18]. After receptor phosphorylation by GRKs, β-arrestins can uncouple receptors from G proteins, promote internalization, scaffold kinase pathways and regulate receptor recycling or degradation [19,20].

Nevertheless, β-arrestin signaling is often oversimplified as they also act as scaffolds and regulators of additional intracellular signaling pathways [21,22]. Early models proposed that opioid-induced analgesia is associated with activation of G protein-dependent mechanisms and the suppression of neuronal excitability, whereas β-arrestin signaling was proposed to contribute to opioid tolerance, dependence, and other adverse effects [23]. For example, administration of morphine to β-arrestin knockout mice produced enhanced and prolonged MOR-mediated antinociception and markedly reduced tolerance [24,25]. While both G protein and β-arrestin signaling pathways can be activated by “balanced” ligands that equally target both pathways (such as morphine), they can also be differentially activated by so-called biased ligands, which stabilize distinct receptor conformations and preferentially activate G protein signaling over β-arrestin recruitment, or vice versa.

TRV 130 (Oliceridine) is a non-peptide MOR agonist initially characterized as G protein-biased, with less gastrointestinal dysfunction and respiratory suppression, relative to morphine, in rodents [26]. It was approved for medical use in the United States in 2020. PZM21 and SR-17018 are other MOR ligands initially characterized as G protein-biased. Both produce prolonged antinociceptive effects in mice; however, subsequent studies demonstrated that PZM21 can induce tolerance and withdrawal, whereas the development of tolerance to SR-17018 appears to depend on the experimental pain model [27,28,29,30,31].

On the other hand, fentanyl, which is one of the most addictive drugs and became one of the leading causes of opioid overdose deaths, exhibits higher efficacy and stronger receptor phosphorylation and β-arrestin recruitment than morphine [32,33].

However, later studies have made this model more nuanced. Current reviews emphasize that many adverse effects may also be G protein-dependent and that the improved pharmacological profiles of some “G protein-biased” ligands may, at least partly, result from their low intrinsic efficacy rather than true pathway selectivity [14,34,35,36]. Those studies indicated that biased agonism alone is insufficient to completely dissociate analgesia from opioid-induced adverse effects. Opioid receptors can also activate kinase cascades, such as the ERK1/2, p38 MAPK, JNK, Src, PKC, and PI3K/AKT pathways, which are relevant not only to acute nociceptive modulation but also to longer-term neuronal plasticity, tolerance, opioid-induced hyperalgesia (OIH), and changes in gene expression [13,37,38,39,40].

**Figure 1:**
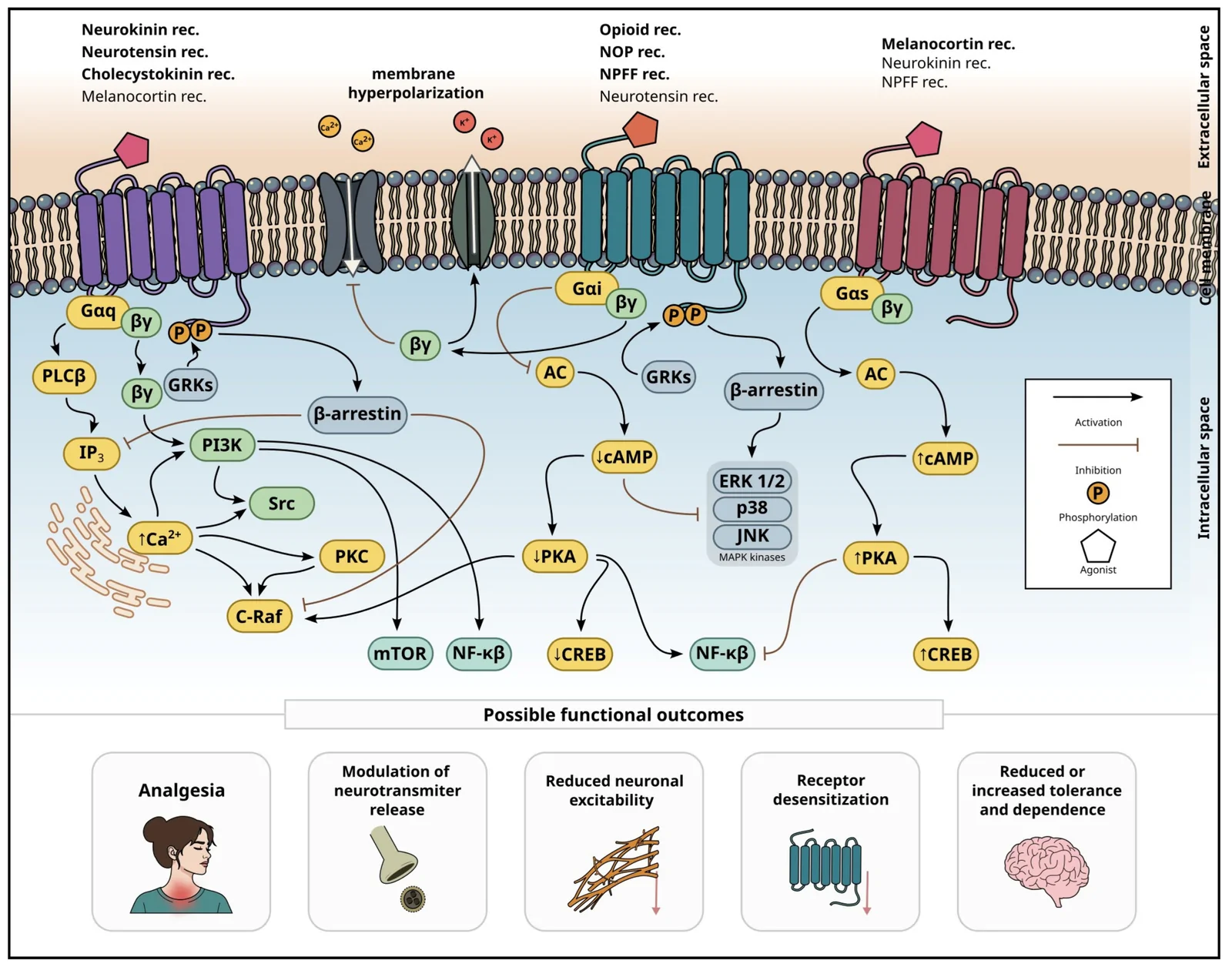
Possible interactions between opioid receptors and other types of GPCRs involved in pain perception.

This caution is especially important for peptide hybrids. Some compounds may show reduced β-arrestin recruitment in a cell line overexpressing the receptor but still produce receptor desensitization, ERK activation, tolerance, or respiratory effects in vivo through other mechanisms. Conversely, efficient receptor internalization and recycling may, in some contexts, preserve opioid responsiveness rather than promote tolerance development. Intracellular signaling profiles and possible interactions between opioid receptors and other types of GPCRs involved in pain perception are summarized in Figure 1.

## 3. NOP Receptor Signaling

The nociceptin/orphanin FQ peptide receptor (NOP) is the fourth member of the opioid receptor family, but a poor target for endogenous opioid peptides. N/OFQ itself has very low affinity at MOR/DOR/KOR, and the classical opioid peptides have correspondingly low affinity at NOP [41]. NOP couples canonically to PTX-sensitive G_i/o_, reduces Ca^2+^ conductance, stimulates inwardly rectifying K^+^ currents, and engages kinase cascade, including PLC, PLA2, PKC and the MAPKs ERK1/2, p38 and JNK, with GRK-mediated receptor phosphorylation driving β-arrestin recruitment, internalization and arrestin-dependent signaling components [41].

NOP receptor activation has a clear modulatory role on MOR-mediated analgesia, tolerance, reward and motor function, and the two receptors are co-expressed and can be functionally and physically coupled throughout the central nervous system [41,42]. Studies described by Bird et al. [43] suggest that MOR and NOP are not pharmacologically independent at the cellular level. In an HEK293 dual-expression system, MOP ligands displaced NOP binding and vice versa, fluorescent dermorphin and N/OFQ probes colocalized by FRET in both HEK and hippocampal tissue, and NOP stimulation produced a rightward shift in the MOR concentration–response curve in both [^35^S]GTPγS binding and cAMP inhibition assays [43].

Single-particle tracking and lipid-raft immunocytochemistry show that morphine, but not buprenorphine, enhances MOR-NOP colocalization on lipid rafts and that morphine stimulates ERK1/2 phosphorylation similarly in MOR-only and MOR + NOP cells, whereas buprenorphine activates ERK1/2 only via the NOP receptor in the co-expression system [44].

In non-human primates, selective NOP agonists produce antinociception without the cardinal MOR liabilities (respiratory depression, itch, and reinforcement) and synergize with MOR-mediated analgesia.

The bifunctional MOR/NOP partial agonists AT-121, BU08028 and BU10038 were also tested in rhesus monkeys. All produced morphine-comparable analgesia without reinforcing effects or respiratory or cardiovascular depression at antinociceptive and even 10–30× higher doses. Interestingly, no measurable physical dependence or tolerance development was observed after repeated administration of tested compounds [45,46,47].

### Opioid/NOP Peptide Hybrids

Several peptide-based strategies have been used to obtain ligands with combined classical opioid and NOP receptor pharmacology. Early bivalent chimeras linked MOP agonist sequences such as dermorphin or YRFB (Tyr-D-Arg-Phe-β-Ala-NH_2_) to the NOP-recognizing hexapeptide Ac-Arg-Tyr-Tyr-Arg-Ile-Lys-NH_2_. The obtained compounds showed affinity for both MOR and NOP receptors and evoked antinociceptive activity after intrathecal administration in rodents [48].

Peptide-based opioid agonist/NOP antagonist hybrids such as KGNOP-type, in which the synthetic opioid tetrapeptide H-Dmt-D-Arg-Aba-β-Ala-NH_2_ is combined with nociceptin or its analogs showed low-nanomolar receptor binding. In in vivo studies, the compounds alleviated allodynia and hyperalgesia in neuropathic pain models, and one hybrid (**13a**, H-Dmt-D-Arg-Aba-β-Ala-Arg-Tyr-Tyr-Arg-Ile-Lys-NH_2_) did not produce significant respiratory depression, compared with an equipotent analgesic dose of morphine [49].

DeNo is a bivalent dermorphin–N/OFQ peptide designed as a mixed MOR/NOP agonist, and in vitro it retained high affinity and agonist activity at both receptors, broadly reflecting the pharmacology of its parent peptides. Functionally, DeNo activated MOP- and NOP-mediated responses in recombinant systems, including G protein-related assays, but its in vivo spinal antinociceptive efficacy was weak, suggesting that simple covalent linkage of two active pharmacophores does not necessarily translate into improved analgesic activity [50].

De101 is a bivalent dermorphin–UFP-101 peptide designed as a mixed MOR agonist/NOP antagonist, in which the dermorphin moiety provides MOP agonism and the UFP-101 moiety provides high-affinity NOP antagonism. In binding and functional assays, De101 showed affinity for both MOP and NOP receptors and stimulated MOP-mediated G protein activation and inhibition of forskolin-induced cAMP formation, but was inactive as a NOP agonist and antagonized N/OFQ responses. Unlike the MOP/NOP agonist DeNo, De101 did not show the same loss of potency in MOP/NOP co-expression systems, suggesting that blocking rather than activating NOP may preserve MOP signaling in this experimental context [43].

The YGGF/RYYRIK-based hybrids BA55, BA61 and BA62 are other examples of opioid–nociceptin chimeric peptides. BA55 showed preferential KOP/NOP interaction and BA62 mainly DOP/KOP-related activity, with the weakest effect in mouse vas deferens. Unexpectedly, BA61, with lack of an N-terminal opioid “message” sequence, behaved as the most potent analog in the functional bioassay, with activity involving mainly DOP and NOP receptors. In [^35^S]GTPγS binding assays, BA55 and BA62 effectively stimulated G protein activation, while BA61 showed weaker/partial agonist-like activity, indicating that linker length, orientation and exposure of the YGGF and RYYRIK motifs strongly determine the pharmacological profile of these opioid/NOP hybrids [51].

The RP-170/RYYRIK chimeras KW-495 and KW-496 were designed by conjugating the cyclic MOP/KOP opioid peptide RP-170 with the NOP ligand fragment RYYRIK-NH_2_, either directly in KW-495 or through a Gly_3_ spacer in KW-496. KW-495 showed a mixed opioid/NOP profile and was selected for in vivo testing, where intrathecal administration produced antinociception in the mouse hot-plate test that was sensitive to both naloxone and the selective NOP antagonist SB-612111, indicating involvement of both classical opioid and NOP receptors. KW-496, in contrast, shifted mainly toward a dual KOP/MOP agonist profile, showing that insertion of the Gly_3_ spacer strongly altered receptor selectivity and functional activity [52]. The structures and activity profiles of opioid/NOP hybrid peptides are summarized in Table 1.

## 4. Neurokinin Receptor Signaling

Neurokinin receptors comprise three tachykinin GPCR subtypes, NK1, NK2 and NK3, which are seven-transmembrane receptors preferentially activated by substance P, neurokinin A and neurokinin B, respectively. They are widely distributed in the central and peripheral nervous systems as well as in gastrointestinal, respiratory, urogenital, vascular, immune, and inflammatory tissues, where they regulate nociceptive transmission, neurogenic inflammation, smooth-muscle contraction, vascular permeability, emesis, stress-related responses and neuroendocrine functions [57].

Substance P is the major tachykinin ligand of NK1 receptor. Its function is to facilitate nociceptive transmission, neurogenic inflammation and central sensitization [58,59].

### 4.1. Neurokinin and Opioid Receptor Cross-Talk

NK1 receptor activation engages G_q/11_-dependent PLC/IP3/Ca^2+^/PKC signaling, but can also recruit G_s_ and β-arrestin-dependent pathways, leading to MAPK/ERK activation, receptor internalization and longer-term transcriptional effects [60].

In contrast, opioid receptors primarily couple to G_i/o_ proteins, inhibit adenylyl cyclase and cAMP formation, reduce voltage-gated calcium-channel activity, activate GIRK potassium channels and modulate kinase cascades such as ERK and AKT [61]. At the spinal level, opioid receptor activation counteracts tachykinin signaling by inhibiting stimulus-evoked substance P release from primary afferent terminals [62]. This presynaptic inhibition limits activation of postsynaptic NK1-positive dorsal horn neurons, thereby reducing amplification of nociceptive signals [58,62].

Anatomical studies show that MOR and NK1 receptors can be co-localized in pain-processing regions, creating a structural basis for receptor cross-talk [59]. Direct receptor-level interaction has also been reported, because MOR and NK1 receptors can form heterodimers that undergo co-internalization and display altered trafficking and resensitization properties [63].

Under sustained opioid receptor stimulation, peripheral nerve injury, or persistent inflammatory input, the physiological inhibitory influence of opioid signaling on nociceptive neurotransmission may be progressively counterbalanced by recruitment of pronociceptive tachykininergic mechanisms. In particular, increased substance P release and/or enhanced neurokinin-1 receptor (NK1R) expression and activation within the spinal dorsal horn have been implicated in central sensitization, reduced opioid analgesic efficacy, OIH, and the development of antinociceptive tolerance. These adaptations may function as part of an endogenous opioid-opposing, or “anti-opioid,” system, in which sustained opioid exposure paradoxically facilitates excitatory nociceptive transmission through substance P/NK1R-dependent mechanisms [58,64,65].

Interestingly, neuropathic injury may impair the ability of MOR activation to inhibit substance P release, which may partly explain the reduced opioid efficacy in some neuropathic pain states [64].

Therefore, NK1 activation often functionally opposes opioid analgesia in spinal pain circuits, whereas opioid receptor activation can suppress NK1-mediated transmission by reducing substance P release. Substance P/NK1 signaling in supraspinal regions may also influence stress, reward, affective pain processing and region-specific analgesic responses [58].

This relationship has encouraged the development of multifunctional ligands combining opioid receptor agonism with NK1 receptor antagonism, aiming to preserve analgesia while limiting substance P-driven pronociceptive plasticity [66].

### 4.2. Opioid/NK Peptide Hybrids

While traditional MOR agonists are the gold standard for treating severe acute pain, their efficacy may be reduced in chronic and neuropathic pain conditions [66]. This loss of efficacy is associated with neuroplastic changes in the central nervous system during prolonged pain states. Specifically, sustained nociceptive input or chronic opioid use can upregulate pronociceptive (pain-enhancing) systems, which may counteract the pain-relieving effects of opioids [67,68].

Because SP can oppose opioid analgesia and contributes to central sensitization (the hyper-excitability of pain pathways), it was hypothesized that hybrid compounds able simultaneously to activate opioid receptors while blocking the NK1 receptor could be highly potent antinociceptive agents [69]. One of representative examples is TY005, a peptidic opioid agonist/NK1R antagonist with the sequence Tyr-D-Ala-Gly-Phe-Met-Pro-Leu-Trp-O-3,5-Bn(CF_3_)_2_. TY005 produced dose-dependent antinociception in uninjured animals and reduced nerve injury-induced thermal and tactile hypersensitivity more effectively than morphine in a rodent neuropathic pain model [70]. TY005 did not produce detectable motor impairment in the rotarod test at antihyperalgesic doses or even at higher doses, and repeated spinal administration did not lead to antihyperalgesic tolerance [70]. These findings support the hypothesis that simultaneous opioid receptor activation and NK1R antagonism may improve analgesic efficacy in neuropathic pain while reducing the tendency toward tolerance development.

Further optimization of opioid/NK1R antagonist peptides focused on conformational restriction, metabolic stability and receptor selectivity. Yamamoto et al. introduced cyclic and topological constraints using penicillamine residues and identified a constrained bifunctional compound with potent opioid agonist activity, marked DOR selectivity and NK1R antagonist activity [71]. Guillemyn and co-workers developed conformationally constrained opioid agonist/NK1R antagonist peptidomimetics based on the opioid pharmacophore Dmt-D-Arg-Aba-Gly and an NK1 antagonist component. These compounds were designed to retain potent opioid activity while incorporating an antagonist element capable of blocking SP/NK1R-mediated pronociceptive signaling. Although some analogs displayed promising in vitro profiles, these studies also demonstrated a key challenge in hybrid design: the two pharmacophores must be arranged so that neither domain sterically or conformationally compromises the other. Linker length, rigidity, charge distribution and C-terminal substitution can markedly influence receptor affinity, functional efficacy and in vivo antinociceptive activity [68].

The potential value of opioid/NK1R antagonist hybrids appears especially under neuropathic pain conditions. Starnowska et al. evaluated bifunctional opioid agonist/NK1R antagonist hybrids in naïve mice and in mice subjected to chronic constriction injury (CCI), a model of neuropathic pain. Biochemical analysis confirmed elevated SP mRNA and protein levels in the lumbar spinal cord of CCI mice, consistent with enhanced pronociceptive tachykininergic tone. In naïve mice, the opioid agonist parent compounds produced acute antinociception more strongly than the hybrids. However, under neuropathic conditions, the opioid ligands showed poor efficacy. In contrast, the best hybrid produced robust and long-lasting alleviation of both tactile and thermal hypersensitivity [66]. This observation is pharmacologically significant because it suggests that NK1R antagonism becomes especially valuable when SP/NK1R signaling is pathologically upregulated.

In another type of opioid/neurokinin hybrid, Spantide and Spantide II were used as NK1R antagonist components derived from substance P. Spantide-like pharmacophores retain structural similarity to SP but contain D-amino acid substitutions and other modifications that convert the tachykinin sequence into an antagonist. This feature makes them attractive for hybridization with opioid peptides, because the C-terminal region of SP and its analogs contains key determinants of NK1R recognition. Wtorek et al. synthesized a series of opioid/neurokinin hybrids in which a cyclic endomorphin-2-derived opioid pharmacophore, Tyr-c[D-Lys-Phe-Phe-Asp]NH_2_, was combined either with SP fragments or with C-terminal fragments of Spantide II [12].

This cyclic opioid fragment displayed mixed MOR/KOR affinity, enhanced enzymatic stability and strong antinociceptive activity. In the Spantide II-containing hybrids, the C-terminal penta- or hexapeptide antagonist fragments were incorporated.

Among these compounds, the opioid agonist/NK1R antagonist hybrid Tyr-[D-Lys-Phe-Phe-Asp]-Asn-D-Trp-Phe-D-Trp-Leu-Nle-NH_2_ produced significant antinociception in the mouse writhing test. Notably, repeated administration did not induce detectable antinociceptive tolerance over the seven-day treatment, and the compound did not reduce stool mass, in contrast to the parent cyclic opioid peptide [12].

Although NK1R antagonism is the most intuitive approach for reducing pronociceptive SP signaling, several studies have shown that substance P-derived agonist pharmacophores can also be incorporated into opioid hybrids with favorable antinociceptive profiles. This apparently paradoxical strategy is based on the observation that SP/NK1R activation can exert different effects depending on dose, anatomical site, receptor population, and temporal context. While high or sustained SP/NK1R activity is generally pronociceptive, controlled coactivation of SP receptors with opioid receptors may maintain opioid responsiveness and reduce tolerance development [72,73].

The prototypical compound in this category is ESP7 (Tyr-Pro-Phe-Phe-Gly-Leu-Met-NH_2_), a substance P/opioid chimeric peptide containing overlapping N-terminal endomorphin-2 and C-terminal SP-derived pharmacophores [73]. Intrathecal administration of ESP7 produced opioid-dependent analgesia in rats, without loss of potency over repeated treatments. Interestingly, when ESP7 was administered together with an SP receptor antagonist, the analgesic effect progressively declined, consistent with tolerance development. Moreover, in morphine-tolerant animals, subsequent ESP7 administration restored a substantial analgesic [73]. These findings suggest that coincident activation of MOR and SP receptors may maintain opioid responsiveness rather than simply amplify acute analgesia.

A closely related chimera, ESP6, differs from ESP7 by a Gly-to-Pro substitution within the overlapping region. ESP6 alone showed reduced analgesic efficacy compared with ESP7, likely due to altered conformational flexibility and lower MOR potency. However, intrahecal co-administration of ESP6 and morphine resulted in prolonged morphine analgesia over a 5-day period. This effect was opioid receptor-dependent and was blocked by an opioid antagonist, naltrexone [74].

Moreover, concurrent NK1R blockade abolished the protective effect of ESP6 against morphine tolerance, supporting the idea that controlled activation of SP/NK1R-dependent mechanisms can participate in anti-tolerance opioid modulation [74].

Wtorek et al. [12] also compared the activities of an opioid/NK1R agonist within the same structural framework. The hybrid compound contained a C-terminal SP hexapeptide fragment, Tyr-[D-Lys-Phe-Phe-Asp]-Gln-Phe-Phe-Gly-Leu-Met-NH_2_, and was directly compared with a Spantide II-derived opioid/NK1R antagonist hybrid. The SP-fragment hybrid retained opioid receptor activity and behaved as an NK1R agonist in calcium mobilization assays. In in vivo studies, it produced significant antinociception in the writhing test, did not induce tolerance and did not cause constipation. Interestingly, no major differences in antinociceptive profile were observed between the NK1R agonist and antagonist hybrids bearing the same opioid fragment [12].

This finding suggests that both NK1R blockade and controlled NK1R activation can, under specific structural and pharmacological conditions, improve opioid-like antinociception. The structures and activity profiles of opioid/NK1 agonist/antagonist hybrid peptides are summarized in Table 2.

## 5. Neurotensin Receptors Signaling

Neurotensin (NT) is an endogenous 13-amino-acid neuropeptide that modulates nociceptive processing predominantly through neurotensin receptor type 1 (NTS1/NTSR1) and type 2 (NTS2/NTSR2), both of which are G protein-coupled receptors. Neurotensin and its receptors are distributed at several levels of pain-processing pathways, including primary sensory structures, the spinal dorsal horn and supraspinal regions. NT-induced antinociception is largely naloxone-insensitive, indicating that neurotensin receptors can engage opioid-independent mechanisms of pain control [86,87].

### 5.1. NTS1 Signaling

NTS1 preferentially couples to G_q/11_, resulting in phospholipase Cβ (PLCβ) activation and hydrolysis of phosphatidylinositol-4,5-bisphosphate (PIP_2_) into inositol-1,4,5-trisphosphate (IP_3_) and diacylglycerol (DAG). IP_3_ promotes Ca^2+^ release from intracellular stores, whereas DAG, together with Ca^2+^, activates conventional protein kinase C (PKC) isoforms. These pathways can influence neuronal excitability, neurotransmitter release, and synaptic transmission and therefore provide potential mechanisms through which NTS1 may modulate nociceptive processing.

However, NTS1 signaling is not restricted to G_q/11_. In recombinant human NTS1-expressing cells, neurotensin also activates G_αi1_, G_αoA_ and G_α13_, modulates cAMP signaling and recruits β-arrestin 1 and β-arrestin 2 [88].

ERK1/2 constitutes an important point of convergence of these pathways. In CHO-K1 cells expressing human NTS1, full ERK1/2 activation required both a pertussis toxin-sensitive G_i/o_-c-Src pathway and a G_q_-PLCβ-DAG-PKC-Raf-1 pathway [88]. Thus, NTS1-induced ERK1/2 phosphorylation results from integrated signaling rather than from a simple linear consequence of G_q_ activation. ERK1/2 signaling may be involved in regulation of neuronal excitability, synaptic plasticity and activity-dependent transcription. Interestingly, persistent spinal ERK activation is frequently associated with sensitization, whereas receptor-restricted and temporally restricted signaling may have different functional consequences. NTS1-dependent ERK1/2 activation should therefore not be regarded as intrinsically pro- or antinociceptive [89].

Following activation, NTS1 can be phosphorylated by GPCR kinases and recruit β-arrestins, promoting receptor desensitization and internalization. The β-arrestins can additionally act as signaling scaffolds, demonstrating that this pathway is not limited to termination of G protein signaling [90,91]. The importance of β-arrestin-dependent NTS1 signaling for pain modulation has recently been demonstrated using SBI-810, an arrestin-biased NTS1 allosteric modulator. SBI-810 produced antinociception in postoperative, inflammatory and neuropathic pain models through mechanisms requiring NTS1 and β-arrestin 2. At the cellular level, it reduced excitatory synaptic transmission and NMDA receptor/ERK signaling in spinal nociceptive neurons, decreased Nav1.7 surface expression and action-potential firing in primary sensory neurons, and attenuated C-fiber responses [92].

These findings support a role for β-arrestin 2-biased NTS1 signaling in reducing peripheral nociceptor excitability and central nociceptive transmission in the investigated experimental models.

### 5.2. NTS2 Signaling

NTS2 was initially characterized as a low-affinity neurotensin-binding receptor but is now recognized as a signaling-competent GPCR involved in pain modulation. Its intracellular signaling is less comprehensively defined than that of NTS1 and should not be assumed to reproduce the canonical NTS1 G_q_/PLC/Ca^2+^ pathway. In CHO cells expressing rat NTS2, neurotensin and NTS2-active ligands induced receptor internalization and ERK1/2 phosphorylation. Inhibition of receptor endocytosis prevented ERK1/2 activation, indicating that NTS2-mediated ERK signaling is dependent on receptor internalization in this experimental system [93]. In the same experimental system, NTS2 stimulation did not elicit a characteristic receptor-dependent Ca^2+^ mobilization response. These findings distinguish NTS2 signaling from the prominent PLC/Ca^2+^ pathway associated with NTS1.

A direct causal link between NTS2-induced ERK1/2 activation and antinociception has not yet been established. Nevertheless, in vivo studies provide strong evidence for the involvement of NTS2 in spinal pain modulation. NTS2 is present in dorsal root ganglia and superficial dorsal horn regions associated with nociceptive processing, and intrathecal administration of NTS2-preferring ligands such as JMV-431 and levocabastine produces antinociceptive effects [94,95].

In the formalin model, these compounds reduced nociceptive behavior and decreased pain-evoked c-Fos expression in superficial dorsal horn neurons, consistent with reduced activation of spinal nociceptive circuits [95]. Several NTS2-selective ligands have additionally produced antinociception without the prominent hypothermia and hypotension often associated with NTS1 activation in preclinical studies, making NTS2 an attractive target for analgesic drug development [96].

Overall, NTS1 and NTS2 appear to modulate pain through partially distinct intracellular mechanisms. NTS1 engages a complex signaling network involving G_q_/PLCβ/Ca^2+^/PKC, additional G protein pathways, ERK1/2 and β-arrestins. Recent evidence supports a role for β-arrestin 2-biased NTS1 signaling in reducing Nav1.7-dependent nociceptor excitability and reduced spinal NMDA/ERK-mediated transmission. In contrast, NTS2 signaling remains less well-characterized but includes internalization-dependent ERK1/2 activation and is associated in vivo with suppression of spinal nociceptive neuronal activity. Further studies are required to determine which intracellular NTS2 pathways are directly responsible for its antinociceptive effects.

### 5.3. Neurotensin Receptor Signaling and Opioid-Independent Analgesia

NT and NT analogs produce analgesic responses that are not abolished by naloxone in several experimental paradigms, indicating that NTSs can directly modulate pain transmission without the activation of opioid receptors [89,95].

NT(8–13) analogs such as JMV2009 and JMV5296 support this concept. JMV2009 activates NTS1 and NTS2, induces sustained p42/p44 MAPK activation and receptor internalization in vitro, and produces antinociceptive activity in acute, tonic, visceral, inflammatory and neuropathic pain models without inducing constipation, tolerance or hypothermia [89]. An NTS2-preferring analog, JMV5296, also produces similar long-lasting antinociception without hypothermia [97].

Interestingly, simultaneous activation of opioid receptors and NTSRs has been shown to enhance antinociception under inflammatory conditions [98]. The neurotensin and opioid systems are functionally interconnected at several levels. Both systems modulate spinal nociceptive input and supraspinal descending pain control. At the cellular level, opioid receptors mainly suppress excitability through G_i/o_-dependent inhibition of cAMP/PKA signaling and regulation of ion channels, whereas NTS1 and NTS2 can engage multiple intracellular pathways, including G_q_-, G_i/o_-, β-arrestin- and ERK-related pathways. As these mechanisms may be complementary, simultaneous activation of opioid and neurotensin receptors could produce additive or potentially synergistic antinociception, possibly reducing the level of opioid receptor activation or the opioid dose required for pain relief.

Experimental data showed that co-administration of morphine with a brain-penetrant NT(8–13) analog improved the analgesic/adverse-effect ratio; at equianalgesic doses, the combination reduced morphine-associated inhibition of gastrointestinal transit compared with morphine alone [98]. Direct receptor-level crosstalk may also occur. Heterodimerization between KOR and NTS1 has been reported to generate a β-arrestin 2-biased signaling pathway [13]. Although the full relevance of this mechanism to analgesia remains to be clarified, it illustrates that opioid and neurotensin receptors can not only interact at the circuit level but may also may influence receptor trafficking, desensitization, MAPK activation and ligand-specific signaling bias.

### 5.4. Opioid–Neurotensin Hybrid Ligands and Intracellular Complementarity

The development of opioid–neurotensin hybrid ligands is based on the same design rationale. These compounds combine opioid pharmacophores with NT-like fragments to engage opioid and neurotensin receptors within a single molecule. PK23 is an example of an opioid–neurotensin hybrid peptide; it produces potent spinal antinociception and shows a more favorable behavioral profile than intrathecally administered morphine, including delayed tolerance development [99].

BNT12, a more recent hybrid peptide containing opioid and NT-like fragments, produces central antinociception in acute, neuropathic, inflammatory, visceral and formalin pain models. Its effects are mediated by MOR and DOR as well as NTS1 and NTS2, and it shows reduced or absent opioid-like side effects in assays of conditioned place preference, withdrawal, locomotor activation, gastrointestinal transit and cardiovascular responses [100].

Opioid–neurotensin hybrids may be useful tools in the development of compounds that can produce analgesia without NTS-mediated hypothermia, hypotension or motor effects. From a signaling perspective, these hybrid ligands may be advantageous because they distribute analgesic efficacy across multiple intracellular systems. The opioid component engages G_i/o_-mediated inhibition of cAMP/PKA signaling, Ca^2+^ influx and neuronal excitability, whereas the neurotensin component can recruit NTS1/NTS2-dependent PLC/PKC, ERK1/2, β-arrestin and receptor-internalization-dependent mechanisms. Such multitarget signaling could contribute to potent antinociception without requiring maximal MOR activation and may potentially be associated with reduced opioid-like adverse effects, although the precise intracellular mechanisms underlying these pharmacological outcomes remain to be established. The structures and activity profiles of hybrid peptides targeting opioid–neurotensin receptors are summarized in Table 3.

## 6. NPFF Receptor Signaling

Neuropeptide FF (NPFF) is an octapeptide (Phe-Leu-Phe-Gln-Pro-Gln-Arg-Phe-NH_2_) which belongs to the RF-amide family of mammalian neuropeptides. Initially isolated from bovine brain tissue, NPFF has become widely recognized as a critical endogenous modulator of nociceptive signaling and opioid homeostasis [106]. The NPFF system consists mainly of two class A G protein-coupled receptors, NPFF receptor 1 (NPFFR1; formerly GPR147) and NPFF receptor 2 (NPFFR2; formerly GPR74), which are activated by NPFF-related RF-amide peptides, including NPFF, neuropeptide AF (NPAF), neuropeptide SF (NPSF/RFRP-1), and RF-amide-related peptide-3 (RFRP-3/NPVF). Both receptors are expressed in central nervous system regions involved in nociceptive processing, including spinal and supraspinal pain-modulatory circuits.

The best-established intracellular mechanism downstream of NPFF receptors is G_i/o_-mediated inhibition of adenylyl cyclase. By reducing cAMP production, NPFF receptor activation may decrease PKA-mediated phosphorylation of ion channels and synaptic proteins, thereby altering neuronal excitability and neurotransmitter release. In pain pathways, cAMP/PKA signaling contributes to peripheral and central sensitization by facilitating voltage-gated Ca^2+^ channels, sodium channels, glutamatergic transmission, and transcriptional regulators such as CREB. Therefore, NPFF receptor-dependent suppression of cAMP/PKA signaling could theoretically exert antinociceptive effects by reducing sensitization-related kinase activity.

However, this interpretation is complicated by the anti-opioid role of the NPFF system. Opioid receptors, particularly MOR, are also G_i/o_-coupled and produce analgesia through inhibition of adenylyl cyclase, suppression of presynaptic Ca^2+^ influx, and activation of K^+^ conductance. NPFF receptor activation can counteract opioid receptor function despite partial convergence on G_i/o_-associated pathways. This suggests that NPFF does not simply mimic opioid receptor signaling; instead, it can reorganize opioid receptor coupling, receptor trafficking, or effector access in a cell-specific manner [107,108].

### 6.1. Regulation of Ca^2+^ Signaling and Voltage-Gated Calcium Channels

NPFF receptor signaling also intersects with Ca^2+^-dependent mechanisms. In neuronal models, NPFF receptor activation can modulate voltage-gated N-type Ca^2+^ channels, which are essential for neurotransmitter release from primary afferent terminals and central nociceptive synapses. Opioid agonists typically inhibit N-type Ca^2+^ channels through G_i/o_ βγ-subunit signaling, thereby reducing release of glutamate, substance P, and CGRP. NPFF receptor activation may antagonize this opioid-mediated inhibition of Ca^2+^ conductance, providing a plausible cellular basis for anti-opioid activity [107,109].

In SH-SY5Y neuroblastoma-derived models expressing NPFF receptors, NPFF receptor activation attenuated opioid-induced inhibition of N-type Ca^2+^ channels and modified opioid receptor coupling to downstream effectors [110,111,112].

Presynaptic Ca^2+^ influx is a central mechanism involved in regulation of nociceptive synaptic transmission, because activation of voltage-gated Ca^2+^ channels, particularly CaV2.2/N-type channels located on primary afferent terminals of the spinal dorsal horn, couples the action of potential invasion to vesicular release of pronociceptive transmitters, including glutamate, substance P and CGRP [113].

Consequently, modulation of presynaptic Ca^2+^ channel activity can markedly influence the strength of excitatory nociceptive input. This mechanism is directly relevant to opioid analgesia, since MOR activation inhibits N-type Ca^2+^ currents in sensory and neuronal models, thereby reducing Ca^2+^-dependent transmitter release [61,114].

The NPFF system appears to intersect with this process at the level of Ca^2+^ channel regulation: NPFF receptor activation can modulate voltage-gated N-type Ca^2+^ channels, and NPFF/NPFF analogs have been shown to counteract opioid-induced inhibition of N-type Ca^2+^ channels in rat spinal ganglion neurons and in NPFF receptor-transfected SH-SY5Y cells [115,116].

Thus, depending on receptor subtype, neuronal population and pain state, NPFF receptor activation may either reduce Ca^2+^-dependent excitatory transmission and contribute to antinociception, or attenuate opioid-mediated inhibition of Ca^2+^ influx, thereby potentially facilitating excitatory neurotransmission and reducing opioid analgesic efficacy. This bidirectional regulation of presynaptic Ca^2+^ signaling provides a plausible cellular explanation for the dual pharmacological profile of NPFF-related peptides, which have been associated with both antinociceptive and anti-opioid/pro-nociceptive effects.

### 6.2. MAPK/ERK Signaling and Receptor Regulation

MAPK/ERK signaling represents another important intracellular pathway linked to NPFF and opioid modulation. In opioid receptor-expressing cells, NPFF analogs can modify opioid-induced ERK phosphorylation, indicating that NPFF-related peptides influence not only second-messenger production but also kinase-dependent signaling dynamics [108]. ERK1/2 activation is highly relevant in nociceptive biology because it regulates synaptic plasticity, transcriptional responses, and long-term changes in dorsal horn excitability. Studies on NPFFR2 have identified agonist-induced receptor phosphorylation sites involved in receptor desensitization and/or internalization, suggesting that kinase-dependent receptor regulation may shape the duration and intensity of NPFF signaling [117,118].

Therefore, it can be assumed that persistent or repeated NPFF receptor activation may produce different outcomes from transient activation, particularly in chronic pain states or during prolonged opioid exposure.

### 6.3. The Neuropeptide FF System as a Target for Multifunctional Opioid Ligands

The NPFF system is an important modulator of opioid tolerance and OIH. Chronic opioid exposure can recruit endogenous NPFF-dependent anti-opioid mechanisms that reduce analgesic efficacy and promote pain hypersensitivity. RF9, originally characterized as an NPFFR1/NPFFR2 antagonist, prevented OIH and the associated development of tolerance in rodents [119]. Subsequent studies, in which more selective pharmacological and genetic approaches were used, confirmed that NPFFR1 contributes to fentanyl-induced hyperalgesia, whereas spinal NPFFR2 can limit opioid analgesia [120,121].

NPFFR1 and NPFFR2 are predominantly G_i/o_-coupled GPCRs, but their anti-opioid effects involve cross-regulation of opioid receptors rather than simple opposition of canonical intracellular signaling. NPFFR2 activation can attenuate MOR-mediated inhibition of voltage-gated Ca^2+^ conductance and induce G_i/o_- and GRK2-dependent heterologous phosphorylation of MOR at Ser377, followed by β-arrestin 2 recruitment and reduced MOR responsiveness in recombinant cellular models [122].

MOR and NPFFR2 can also physically associate, and NPFFR2 activation alters MOR membrane organization and mobility [111,123]. In addition, NPFFR2 activation has been linked to increased CGRP expression and release and to hyperalgesia, although this represents a broader pronociceptive mechanism rather than a specifically established pathway of OIH [124].

These findings provide a rationale for bifunctional MOR agonist/NPFFR antagonist ligands. KGFF09 combines G protein-biased MOR agonism with NPFFR antagonism and produces potent antinociception with markedly reduced development of hyperalgesia, tolerance and physical dependence during repeated treatments [125].

More recently developed MOR agonist/NPFFR antagonist hybrids have similarly produced potent antinociception with reduced opioid-related adverse effects in preclinical models [126]. The opioid component suppresses nociceptive transmission through G_i/o_-dependent inhibition of adenylyl cyclase and voltage-gated Ca^2+^ channels, whereas NPFFR antagonism may limit the recruitment of NPFF-dependent anti-opioid adaptations. Opioid/NPFFR agonist hybrids represent an alternative strategy. EN-9, BN-9, DN-9 and related cyclic or stapled analogs have produced potent and prolonged antinociception, in several studies, with limited tolerance and other opioid-related adverse effects [127,128,129,130,131]. However, NPFFR blockade can enhance the acute antinociceptive effect of DN-9, indicating that NPFFR activation may partially oppose its opioid-mediated analgesia [129].

Thus, NPFFR agonism should not be regarded as intrinsically analgesic or protective against tolerance. Overall, both opioid agonist/NPFFR antagonist and opioid/NPFFR agonist hybrids are promising preclinical strategies, although the available evidence more directly links NPFFR antagonism to the prevention of OIH and tolerance in certain experimental models, while the intracellular mechanisms underlying the favorable pharmacological profiles of NPFFR agonist hybrids remain to be fully elucidated. The structures and activity profiles of opioid/neuropeptide FF hybrid peptides are summarized in Table 4.

## 7. Cholecystokinin Receptor Signaling

Cholecystokinin (CCK) is a biologically active peptide that functions both as a gastrointestinal hormone and as a neuromodulator in the central and peripheral nervous systems. Its biological effects are mediated mainly by two class A G protein-coupled receptors: cholecystokinin receptor type 1 (CCK1R/CCKAR), originally characterized as the peripheral “A” receptor, and cholecystokinin receptor type 2 (CCK2R/CCKBR), historically referred to as the central “B” receptor. Although both receptor subtypes may participate in nociceptive modulation, CCK2R is the receptor most consistently implicated in pain facilitation, anti-opioid activity, opioid tolerance and OIH [142,143,144,145].

CCK1R and CCK2R are primarily coupled to G_q/11_ proteins. Upon agonist binding, receptor activation stimulates phospholipase Cβ (PLCβ), which hydrolyses phosphatidylinositol 4,5-bisphosphate into inositol 1,4,5-trisphosphate (IP_3_) and diacylglycerol (DAG). IP_3_ promotes Ca^2+^ release from intracellular stores, whereas DAG activates protein kinase C (PKC). Thus, the classical intracellular signaling axis activated by CCK receptors can be summarized as G_q_/PLCβ/IP_3_/DAG/Ca^2+^/PKC [143].

This pathway is highly relevant to nociceptive processing, as intracellular Ca^2+^ and PKC regulate neuronal excitability, neurotransmitter release, receptor phosphorylation, membrane-channel function and activity-dependent plasticity [143,144,145].

In addition to G_q/11_-dependent signaling, CCK receptors can activate several kinase-associated pathways, including ERK1/2, JNK, p38 MAPK, PI3K/Akt, Src-family kinases, focal adhesion kinase-related pathways and other tyrosine kinase-dependent signaling modules [143,144].

### 7.1. Ca^2+^-Dependent Signaling and Nociceptive Excitability

The CCK receptor-induced increase in intracellular Ca^2+^ is one of the most important cellular events linking receptor activation to altered neuronal function. Therefore, CCK receptor-dependent Ca^2+^ mobilization provides a plausible mechanism through which CCK may facilitate nociceptive transmission, especially under conditions of persistent peripheral input, inflammatory sensitization, nerve injury or prolonged opioid exposure [143,144,145].

Especially, CCK2R activation has been associated with neuronal depolarization and activation of Ca^2+^-dependent intracellular cascades, which may enhance neuronal responsiveness to nociceptive input, particularly through interaction with other neuromodulatory systems, e.g., the opioid system [144,145].

### 7.2. PKC, MAPK and PI3K/Akt Signaling as Modulators of Pain-Related Plasticity

PKC is a central downstream effector of CCK receptor activation. In nociceptive circuits, PKC can phosphorylate ion channels, glutamatergic receptors and other signaling proteins that regulate neuronal excitability and synaptic efficacy. Therefore, CCK receptor-mediated PKC activation may contribute to increased responsiveness of pain-processing neurons and to the maintenance of activity-dependent plasticity. This mechanism is consistent with the broader role of G_q_-coupled receptor signaling in enhancing excitatory neurotransmission and sensitization-related intracellular responses [143,144,145].

CCK receptors may also activate MAPK pathways, including ERK1/2, JNK and p38 MAPK. However, the evidence directly connecting CCK receptor-triggered MAPK activation to pain facilitation is not equally strong for all cell types and pain models [143,144,145].

Similarly, CCK receptor-associated PI3K/Akt signaling may participate in cellular plasticity, survival signaling, receptor regulation and modulation of synaptic function. Although PI3K/Akt is not the most specific hallmark of CCK-mediated nociception, it belongs to the broader network of intracellular kinase pathways activated downstream of CCK and may contribute to pain-related adaptations, although the specific involvement of Akt in CCK-mediated nociceptive signaling remains to be fully established [143,144].

### 7.3. CCK2R as an Endogenous Anti-Opioid System

A subset of brainstem neurons co-expresses MOR and CCK2R, providing an anatomical and cellular substrate for functional opioid–CCK interactions in descending pain control [146]. Cholecystokinin octapeptide, CCK-8, has been shown to antagonize opioid analgesia mediated by MOR and KOR in the rat spinal cord [147]. Moreover, the blockade of CCK receptors with antagonists such as proglumide potentiated morphine analgesia in preclinical models, supporting the hypothesis that CCK-evoked signaling can limit opioid antinociception [148]. Selective CCK2R antagonists like L-365,260 or CI988 enhanced morphine analgesia and prevented morphine tolerance in rats [149,150]. In a rat model of peripheral neuropathic pain, co-administration of L-365,260 prevented tolerance to the antinociceptive effects of morphine [151]. Therefore, CCK2R-mediated G_q_/Ca^2+^/PKC and kinase signaling may functionally counterbalance opioid-induced neuronal inhibition [143,147].

Since opioid receptors are primarily G_i/o_-coupled receptors that inhibit adenylyl cyclase, reduce Ca^2+^ influx and suppress transmitter release, CCK2R-mediated G_q_/Ca^2+^/PKC and kinase signaling may functionally counterbalance opioid-induced neuronal inhibition [143,147]. A major site of CCK-mediated pain facilitation is the rostral ventromedial medulla (RVM), a key brainstem structure controlling descending modulation of spinal nociceptive transmission. CCK in the RVM contributes to opioid-induced hyperalgesia and antinociceptive tolerance. Xie and colleagues demonstrated that CCK signaling in the RVM is involved in the pronociceptive consequences of sustained opioid exposure, supporting the concept that CCK can recruit descending facilitatory pathways that oppose opioid analgesia [152]. It was also reported that activation of descending pain-facilitatory pathways from the RVM by CCK elicits an increase in spinal prostaglandin E_2_ levels. This provides a mechanistic link between supraspinal CCK2R activation and spinal sensitization: CCK signaling in the brainstem may increase descending facilitation, which in turn promotes spinal prostaglandin signaling and enhances nociceptive processing [153].

Sustained opioid administration may recruit endogenous CCK signaling as a compensatory anti-opioid mechanism. Once activated, CCK2R-dependent signaling may increase excitatory Ca^2+^/PKC and kinase-dependent pathways, strengthen descending pain facilitation and reduce the net inhibitory effect of opioid receptor activation, which may contribute to antinociceptive tolerance and opioid-induced hyperalgesia [147,149,150,151,152]. This interaction was also observed in mice with deletion of the CCK2R gene, in which it resulted in upregulation of components of the endogenous opioid system [154].

These findings support the view that CCK2R is not only a pain-facilitating receptor, but also a homeostatic modulator of endogenous opioid signaling and opioid responsiveness [144,145,154]. However, clinical translation has been difficult. Although proglumide potentiated morphine analgesia in an experimental human pain study, later clinical work failed to demonstrate potentiation of morphine analgesia by proglumide in a randomized double-blind postoperative patient-controlled analgesia setting [155,156].

These mixed outcomes suggest that the therapeutic benefit of CCK receptor antagonism may depend on antagonist selectivity, CNS penetration, pain etiology, opioid dose, timing of administration and the degree to which endogenous CCK signaling is recruited in a given clinical condition.

Another therapeutic direction is the development of multifunctional analogs that combine opioid receptor agonist pharmacophore with CCK2R antagonist pharmacophore. This strategy remains less developed than opioid–neurokinin or opioid–NPFF hybrid pharmacology, but may be justified by the involvement of CCK2R signaling in the modulation of opioid analgesia, morphine tolerance, opioid-induced hyperalgesia and descending pain facilitation [146,147,149,150,151,152,153]. The structures and activity profiles of opioid agonist/CCK2R antagonist hybrid peptides are summarized in Table 5.

## 8. Melanocortin Receptor-Associated Intracellular Signaling

The melanocortin system is composed of peptide ligands derived from proopiomelanocortin (POMC), including α-, β- and γ-melanocyte-stimulating hormone (MSH) and adrenocorticotropic hormone (ACTH), and five melanocortin receptors, MC1R-MC5R. These receptors belong to the class A G protein-coupled receptor (GPCR) family and are associated with G_s_-mediated stimulation of adenylyl cyclase, intracellular cAMP accumulation and activation of protein kinase A (PKA). Although MC4R is most widely recognized for its role in regulating energy homeostasis and feeding behavior, increasing evidence indicates that it also contributes to the control of glucose metabolism, stress responses, reward processing, sexual behavior, inflammatory modulation, and nociceptive signaling [165,166].

Melanocortin receptor signaling is not limited to the G_s_/cAMP/PKA axis. Depending on receptor subtype, ligand structure, receptor expression level and accessory proteins, melanocortin receptors may also engage ERK1/2, p38 MAPK, JNK, PI3K/Akt, Ca^2+^-dependent signaling and β-arrestin-associated regulatory mechanisms [165,166].

Among melanocortin receptor subtypes, MC4R has the strongest preclinical association with pronociceptive signaling, especially in neuropathic pain. Pharmacological studies in rodent nerve-injury models indicate that blockade of the spinal or peripheral melanocortin system reduces mechanical allodynia and thermal hyperalgesia, whereas melanocortin receptor agonists can enhance nociceptive sensitivity [167,168,169,170,171].

Nevertheless, frequently used ligands such as SHU9119 and MTII are not absolutely selective for MC4R and can interact with both MC3R and MC4R. Therefore, the available data should be more broadly interpreted, as in some experimental settings mixed MC3R/MC4R melanocortin signaling has contributed to pathological pain sensitization [168,169,170,171,172].

### 8.1. cAMP/PKA-Dependent Mechanisms

The classical intracellular pathway activated by melanocortin receptors involves G_s_-dependent stimulation of adenylyl cyclase, increased intracellular cAMP, and activation of PKA [165,166]. PKA phosphorylates multiple downstream substrates, including ion channels, transcription factors and proteins involved in synaptic regulation. In neuronal systems, this pathway can alter membrane excitability, neurotransmitter release and activity-dependent plasticity. In immune and glial cells, cAMP/PKA signaling may suppress pro-inflammatory transcriptional programs, partly through inhibition of NF-κB-dependent responses and promotion of anti-inflammatory mediators [173,174].

Nevertheless, melanocortin receptor modulation cannot be classified as uniformly pro- or antinociceptive, as activation of MC1R or MC3R in immune and inflammatory cells is generally associated with anti-inflammatory and pro-resolving responses, whereas MC4R activation within pain-processing neuronal circuits may facilitate nociception [173,174,175,176,177,178,179].

### 8.2. MC4R, P38 MAPK and Neuropathic Pain

A substantial body of preclinical evidence links MC4R signaling with MAPK activation in neuropathic pain. In a rat chronic constriction injury model, Chu and colleagues reported increased MC4R expression and elevated phosphorylated p38 MAPK in the lumbar spinal cord. Intrathecal administration of the MC4R antagonist HS014 reduced thermal hyperalgesia and prevented p38 MAPK activation, whereas pharmacological inhibition of p38 MAPK reduced hyperalgesia without preventing MC4R upregulation [172].

These findings support a model in which p38 MAPK acts downstream of MC4R activation in the spinal cord during neuropathic pain.

A related study implicated MC4R-dependent p38 MAPK activation in dorsal root ganglia after chronic constriction injury [175].

This suggests that MC4R may contribute to neuropathic hypersensitivity not only at the spinal dorsal horn level but also within primary sensory neurons. Since p38 MAPK is known to regulate the production of inflammatory mediators, transcriptional plasticity, and nociceptor sensitization, MC4R-dependent p38 activation provides a plausible intracellular route through which melanocortin signaling may amplify neuropathic pain. However, because p38 MAPK can be activated in neurons, microglia, astrocytes and peripheral sensory neurons, the cellular source of this signaling may differ across pain models.

### 8.3. MC4R and JNK/c-Jun Signaling

MC4R has also been linked to JNK signaling in neuropathic pain. Zhao and colleagues demonstrated that chronic constriction injury increased MC4R expression and activated the JNK pathway, as indicated by increased JNK phosphorylation and c-Jun expression. Pharmacological blockade of MC4R with HS014 attenuated behavioral hypersensitivity and reduced activation of JNK-related signaling [180]. JNK/c-Jun signaling participates in stress-induced transcriptional responses, glial activation and long-term nociceptive plasticity. Therefore, MC4R-associated JNK signaling may contribute to the maintenance of neuropathic pain by promoting persistent changes in pain-related gene expression and cellular excitability.

### 8.4. PI3K-Dependent Modulation of Sensory Neuronal Excitability

Beyond transcriptional and MAPK-dependent mechanisms, MC4R can directly influence sensory neuron excitability through ion channel modulation. In trigeminal ganglion neurons, α-MSH-mediated activation of MC4R suppresses transient outward A-type K^+^ currents through a PI3K-dependent mechanism [181]. A-type K^+^ currents normally oppose membrane depolarization and limit repetitive firing. Their inhibition can lower the threshold for action potential generation, increase neuronal firing and enhance mechanical pain sensitivity [181].

Thus, MC4R activation may contribute to nociceptive sensitization not only through inflammatory or transcriptional pathways but also through acute modulation of membrane excitability. This mechanism provides a direct cellular explanation for melanocortin-mediated pain facilitation. If MC4R activation reduces potassium-channel-mediated repolarizing control in sensory neurons, nociceptive neurons become more responsive to peripheral stimuli, thereby amplifying nociceptive input to the spinal cord.

### 8.5. Functional Interaction Between Melanocortin and Opioid Systems

The formalin test has provided additional evidence for the contribution of spinal melanocortin signaling to nociceptive sensitivity. The intrathecal administration of the melanocortin receptor agonist MTII increased nociceptive behavior in both phases of the mouse formalin test. In contrast, melanocortin receptor antagonists, including SHU9119, HS014 and JKC-363, as well as endogenous Agouti-related protein, reduced nociception in the late inflammatory phase [182].

These findings support the concept that spinal melanocortin receptor activation can facilitate inflammatory nociceptive processing, particularly during the second phase of the formalin response, which reflects inflammatory and central sensitization-related mechanisms. It can be concluded that activation of spinal melanocortin signaling can increase nociceptive sensitivity, whereas blockade of melanocortin receptors can reduce inflammatory pain-like behavior in this model.

MC1R is best known for its role in pigmentation, but it is also expressed in immune and non-melanocytic cells and contributes to inflammatory regulation. Activation of MC1R by α-MSH increases cAMP/PKA signaling and can inhibit NF-κB-dependent transcription of pro-inflammatory mediators. This anti-inflammatory profile includes reduced production of cytokines and inflammatory mediators and increased protective or pro-resolving responses [173,174]. Delaney and colleagues showed that MC1R is involved in acute pain and inflammatory pain, but not neuropathic pain, in the studied experimental models, with sex-dependent effects [176]. The relationship between MC1R, melanocortins and opioid tone is further supported by studies of red-hair-associated MC1R loss-of-function biology. In red-haired mice, reduced MC1R signaling in melanocytes decreases circulating melanocortin tone and indirectly reduces MC4R-mediated opposition to MOR signaling in the periaqueductal gray. This mechanism was proposed to contribute to altered nociceptive thresholds associated with red hair phenotypes [177].

These data provide an important physiological link between peripheral melanocortin biology, central MC4R signaling and endogenous opioid-mediated pain control.

MC3R was not as extensively studied in pain models as MC4R, but it has important anti-inflammatory functions that may be relevant to inflammatory pain. MC3R is expressed in immune cells, including macrophages, and melanocortin signaling through MC3R can reduce inflammatory cell recruitment and suppress cytokine-driven inflammatory responses [178,179].

One of the potential therapeutic strategies emerging from melanocortin pain biology is MC4R antagonism. In rodent neuropathic pain models, melanocortin receptor antagonists reduce mechanical allodynia and thermal hyperalgesia after spinal, central or peripheral administration [167,168,169,170,171].

Therefore, bifunctional opioid agonist/MC4R antagonist ligands gained the scientists’ interest as a strategy to engage two complementary mechanisms involved in pain modulation. These compounds are designed to combine opioid receptor-mediated inhibition of nociceptive transmission with blockade of MC4R-mediated pronociceptive signaling. In preclinical neuropathic pain models, hybrid compounds produced potent antinociceptive effects [183,184]. Later work on bifunctional opioid/melanocortin peptidomimetics further demonstrated that linker type and linker length influence pharmacological activity and efficacy in neuropathic pain models [9].

Although the strategy of combining an opioid agonist pharmacophore with an MC4R antagonist pharmacophore appears promising, the pharmacological activity of those compounds is not fully determined. Their pharmacokinetic properties, blood–brain barrier penetration, receptor selectivity, long-term safety, tolerance liability, respiratory effects and abuse potential require further systematic evaluation before translational conclusions can be drawn. The structures and activity profiles of opioid/melanocortin hybrid peptides are summarized in Table 6.

## 9. Discussion

### 9.1. The Rationale for Pharmacological Hybridization

The development of opioid peptide hybrids containing additional pharmacophores directed toward non-opioid peptide receptors represents a rational strategy for improving analgesic efficacy while reducing the limitations associated with classical opioid receptor activation. Conventional MOR agonists remain highly effective analgesics, but their clinical utility is limited by tolerance, OIH, constipation, respiratory depression, physical dependence, and abuse liability. In this context, multifunctional peptide ligands should not be regarded simply as “stronger opioids,” but rather as pharmacological tools designed to redistribute analgesic efficacy across several pain-modulatory systems. By combining an opioid pharmacophore with a second peptide or peptidomimetic motif, these compounds may either activate an additional antinociceptive pathway or block an endogenous pronociceptive/anti-opioid mechanism that limits opioid efficacy [70,99,100,125,126].

This concept is particularly significant as persistent pain is not driven by a single receptor system. Neuropathic, inflammatory, visceral, and cancer-related pain states involve plastic changes in primary afferent neurons, spinal dorsal horn neurons, glial cells, descending modulatory pathways, and supraspinal circuits. These changes include altered opioid receptor function, increased excitatory neuropeptide signaling, enhanced kinase-dependent sensitization, recruitment of anti-opioid systems, and neuroimmune activation. Therefore, hybrid opioid peptides targeting NOP, neurokinin, neurotensin, NPFF, cholecystokinin, melanocortin, and other GPCRs may be therapeutically advantageous when their second pharmacophore addresses a defined biological limitation of opioid pharmacology [100,125,126,150,151,183,184].

### 9.2. Dual Analgesic Activation: NOP and Neurotensin Hybrids

An important direction is the design of opioid/NOP receptor hybrids. The nociceptin/orphanin FQ peptide receptor signals mainly through G_i/o_-dependent mechanisms similar to classical opioid receptors, but its functional role in pain depends on the anatomical site of action and the specific pain state. The NOP receptor modulation may produce antinociception, modify opioid reward, alter tolerance development, or even oppose classical opioid effects. Some compounds have been successfully designed as opioid receptor agonist/NOP receptor antagonist ligands for the dual treatment of acute and neuropathic pain, whereas others, such as KGNOP1, act as bifunctional opioid/NOP receptor agonists with robust antinociceptive activity in neuropathic pain models [49,55]. These studies suggest that the optimal NOP component may not always be simple full agonism; rather, future compounds should be optimized for pain-state-specific efficacy and well-defined NOP intrinsic activity.

Opioid–neurotensin hybrids represent a complementary strategy based on the ability of neurotensin receptors to mediate opioid-independent or opioid-sparing antinociception. Neurotensin receptors (NTS1 and NTS2) are coupled to intracellular pathways involving G_q_/PLC/Ca^2+^/PKC cascades, G_i_/_o_-dependent signaling, and ERK1/2 activation. NTS2-preferring activation is especially attractive because it preserves antinociceptive efficacy while limiting NTS1-associated adverse effects, such as hypothermia and hypotension. Opioid–neurotensin hybrids such as PK23 and BNT12 support this thesis. PK23 produced long-lasting spinal antinociception and delayed tolerance development in preclinical studies, while BNT12 produced potent central antinociception with a reduced side-effect profile in several rodent assays [99,100]. These data demonstrate that neurotensin pharmacophores can act as independent analgesic modules capable of expanding the therapeutic window of opioid-based peptides.

### 9.3. Antagonizing Pronociceptive and Anti-Opioid Systems

Alternatively, hybrids can be designed to block systems that actively oppose opioid analgesia. Opioid/neurokinin hybrids constitute one of the most developed examples of this “opioid plus anti-pronociceptive blockade” strategy. Substance P/NK1 receptor signaling is strongly associated with nociceptive transmission, central sensitization, and opioid-opposing neuroadaptations. Opioid agonist/NK1 receptor antagonist hybrids, such as TY005, are designed to preserve opioid-mediated inhibition while blocking tachykininergic pronociceptive signaling. TY005 significantly attenuated pain in preclinical models, with reduced tolerance development compared with classical opioid treatment [70].

However, the neurokinin field illustrates an important nuance: some opioid/substance P-derived NK-1 agonist chimeras may modulate tolerance through controlled co-activation rather than receptor blockade. This indicates that the second pharmacophore should not be classified as intrinsically “beneficial” without considering receptor efficacy, dose, anatomical site, and pain state.

The neuropeptide FF (NPFF) system provides another highly relevant target for blocking endogenous anti-opioid mechanisms. NPFF receptors participate in opioid modulation and are heavily implicated in OIH and tolerance. Therefore, several advanced hybrids combine MOR agonism with NPFF receptor antagonism. KGFF09, a bifunctional biased MOR agonist/NPFF receptor antagonist, showed potent antinociceptive activity with improved acute and chronic side-effect profiles [125]. More recently, optimized DP-series analogs produced robust antinociception with reduced adverse effects in mice [126].

These findings support the broader hypothesis that blocking anti-opioid peptide systems may be as therapeutically important as activating additional analgesic receptor pathways.

Cholecystokinin (CCK) and melanocortin (MC4R) receptor pharmacology support a similar conceptual framework. CCK-2 receptors actively oppose opioid analgesia and contribute to morphine tolerance. While selective CCK-2 receptor antagonists (e.g., CI988 and L-365,260) prevent morphine tolerance development, fully optimized opioid/CCK peptide hybrids with comprehensive in vivo safety profiling remain a plausible but less mature strategy [150,151].

Opioid agonist/MC4R antagonist hybrids have recently been shown to produce potent antinociceptive effects in mouse and rat neuropathic pain models by combining opioid receptor-mediated inhibition with the simultaneous blockade of melanocortin-driven pain facilitation [183,184].

Additional receptor systems may further expand this framework. For example, opioid/cannabinoid bifunctional ligands (e.g., OCP002) have demonstrated potent antinociception with minimized side effects in mouse models [188]. Development and optimization of opioid hybrids simultaneously targeting bradykinin receptors or neuropeptide Y (NPY) receptors, particularly Y1 and Y2, also represent attractive future targets, although those compounds remain less developed than their NK1 or NPFF counterparts [189,190,191,192].

### 9.4. Preclinical Limitations and Future Directions

Future development of opioid peptide hybrids should prioritize several pharmacological principles. First, receptor efficacy, not just affinity, must be optimized (e.g., utilizing partial MOR agonism, NTS2 preference, or NPFF antagonism). Second, intracellular signaling bias (G protein activation vs. β-arrestin recruitment) should be systematically characterized, as it heavily influences the development of the adverse-effect profile. Third, linker structure (length, flexibility, and metabolic stability) is critical for ensuring both pharmacophores retain biological activity. Finally, pharmacokinetics must be considered early, as systemic utility is often limited by enzymatic degradation and restricted blood–brain barrier penetration.

The main limitation within the development of opioid hybrid peptides is that favorable side-effect profiles are frequently based on incomplete preclinical assessments, while their enzymatic stability and pharmacokinetic properties remain insufficiently characterized. Proteolytic degradation may limit the therapeutic efficacy of peptide compounds due to limited bioavailability and duration of action [193]. For example, EMs are susceptible to degradation by dipeptidyl peptidase IV and aminopeptidase M, whereas neurotensin is inactivated by zinc metallopeptidases [194,195]. Structural optimization strategies, including cyclization, incorporation of D- or non-natural amino acids and introduction of other modifications such as glycosylation, can improve resistance to enzymatic degradation [196]. However, enhanced in vitro stability does not necessarily translate into favorable in vivo pharmacokinetics or prolonged analgesic efficacy. Hybridization itself may influence susceptibility to enzymatic cleavage by altering the accessibility of protease-sensitive peptide bonds, as suggested by studies of opioid–neurotensin hybrids [105]. Further structural modifications may improve enzymatic stability, as demonstrated by opioid–neurotensin peptidomimetics exhibiting half-lives exceeding 48 h in rat plasma in vitro and the disulfide-cyclized opioid–NK1R hybrid TY038, which showed a plasma half-life exceeding 24 h in vitro [83,105]. Glycosylation also enhanced the stability of an opioid–NK1R hybrid, with 70 ± 9% remaining intact after 24 h in rat plasma [82]. Nevertheless, enzymatic stability remains insufficiently characterized for many opioid hybrid peptides reported to date, limiting our understanding of their susceptibility to proteolytic degradation and its potential impact on pharmacological efficacy. Therefore, comprehensive evaluation of both metabolic stability and opioid-related adverse effects is essential for assessing the therapeutic potential of these multifunctional ligands.

In parallel, a more detailed understanding of how intracellular signaling pathways engaged by individual pharmacophores interact within a single hybrid molecule is essential to determine how signaling cross-talk, pathway convergence and functional bias contribute to overall analgesic efficacy and adverse-effect profiles. Reduced tolerance in a tail-flick or von Frey paradigm does not guarantee the absence of respiratory depression, reward liability or physical dependence. Future studies should therefore combine comprehensive assessment of enzymatic stability and pharmacokinetics with overall characterization of receptor signaling and standardized side-effect evaluations, including respiratory function, gastrointestinal transit, reward/aversion and the consequences of repeated administration.

## 10. Conclusions

Opioid peptide hybrids targeting additional neuropeptide or peptide-sensitive receptor systems provide a promising framework for the development of next-generation analgesics. Their primary value lies not simply in increased potency, but in their capacity to functionally reshape pain-related signaling networks. Although most of these compounds remain in experimental stages, they provide a coherent medicinal chemistry strategy for complex pain states where single-target opioid pharmacology often remains insufficient.

## Figures and Tables

**Table 1:** Opioid/NOP hybrid structures and activity profiles.

Compound/Family	Structure or Design	Main Profile	Key Reference
**Dermorphin–Ac-RYYRIK hybrids**	Dermorphin, Tyr-D-Ala-Phe-Gly-Tyr-Pro-Ser-NH_2_, or YRFB, Tyr-D-Arg-Phe-βAla-NH_2_, linked to the ORL1/NOP ligand Ac-Arg-Tyr-Tyr-Arg-Ile-Lys-NH_2_ through spacers.	Early MOP/NOP chimeras. Chimeric peptide 2, containing dermorphin and Ac-RYYRIK-NH_2_ connected by a long spacer, showed the highest affinity for both receptors; i.t. administration produced potent antinociception in mice.	[48,53]
**KGNOP1/compound 13a**	H-Dmt-D-Arg-Aba-β-Ala-Arg-Tyr-Tyr-Arg-Ile-Lys-NH_2_. It links the opioid pharmacophore H-Dmt-D-Arg-Aba-β-Ala-NH_2_/KGOP01-like motif with the NOP-related RYYRIK (-Arg-Tyr-Tyr-Arg-Ile-Lys-NH_2_) sequence.	Initially described as an opioid agonist/NOP antagonist hybrid in vitro, but later in vivo studies support participation of both MOR and NOP mechanisms. It showed potent antinociception in acute, inflammatory and neuropathic pain models, with reduced respiratory depression and reduced rewarding/withdrawal-like liabilities compared with morphine.	[49,54,55,56]
**DeNo: Dermorphin–N/OFQ**	Bivalent peptide made by linking the MOP agonist dermorphin with the endogenous NOP agonist N/OFQ.	Designed as a mixed MOR/NOP agonist. In vitro, it behaved broadly like the parent pharmacophores at MOP and NOP, but its spinal antinociceptive effect was weak in the tested rat model.	[50]
**De101: Dermorphin–UFP-101**	Dermorphin linked to UFP-101, a NOP antagonist peptide.	Designed as a MOR agonist/NOP antagonist bivalent ligand; used mainly to study MOP-NOP receptor interaction rather than as an optimized analgesic lead.	[43]
**BA55**	Tyr-Gly-Gly-Phe-Gly-Gly-Gly-Arg-Tyr-Tyr-Arg-Ile-Lys -NH_2_. Opioid message sequence linked by -Gly_3_- to RYYRIK.	MOR/DOR/KOR/NOP mixed ligand, built from a minimal endogenous opioid-like N-terminal motif and the Ac-RYYRIK-derived NOP-recognition sequence.	[51]
**BA62**	Tyr-Gly-Gly-Phe-Arg-Tyr-Tyr-Arg-Ile-Lys-NH_2_. Direct YGGF-RYYRIK fusion without Gly_3_ spacer.	Highest affinity for KOR, moderate for NOP, weak for MOR/DOR. Highest G protein efficacy of the series. The absence of the spacer changed receptor activity, illustrating that linker architecture strongly affects opioid/NOP hybrid pharmacology.	[51]
**BA61**	Ac-Arg-Tyr-Tyr-Arg-Ile-Lys-Gl-Gly-Gly-Tyr-Gly-Gly-Phe-Leu-OH. Reverse orientation: NOP-related RYYRIK at the N-terminus, Gly_3_ linker, opioid-like YGGFL at the C-terminus.	DOR/NOP ligand from the Erdei series. The different orientation and terminal chemistry produced a distinct mode of action compared with BA55 and BA62.	[51]
**KW-495**	EM-2 cyclic analog with MOR/KOR affinity: Tyr-c[D-Lys-Phe-Phe]Asp-NH_2_, directly conjugated to -Arg-Tyr-Tyr-Arg-Ile-Lys-NH_2_.	Mixed MOR/KOR/NOP agonist with low-nanomolar affinity. In mice, i.t. KW-495 produced antinociception blocked by both naloxone and the NOP antagonist SB-612111, supporting opioid and NOP involvement.	[52]

**Table 2:** Opioid/NK1 hybrids containing NK1 agonist or substance-P-like pharmacophores.

Compound/ Family	Structure or Design	Main Profile	Key Reference
**Endomorphin-1/substance P derivatives**	Three N-terminal amino acids of endomorphin-1 conjugated to the C-terminal SP fragments: SP_8–11_ (-Phe-Gly-Leu-Met-NH_2_) or SP_7–11_ (Phe-Phe-Gly-Leu-Met-NH_2_). Some analogs were N-terminally lipidated with C_10_ lipoamino acid (C10LAA).	Lipidation improved metabolic stability and membrane permeability. Compound **5** with highest membrane permeability showed potent MOR affinity (K_i_ = 3.87 ± 0.51 nM) and dose-dependent MOR agonist activity (IC_50_ = 45 ± 13 nM).	[75]
**ESP7** **Endomorphin-2/substance P derivatives**	Tyr-Pro-Phe-Phe-Gly-Leu-Met-NH_2_ (YPFFGLM-NH_2_). The N-terminal part corresponds to endomorphin-2, while the C-terminal segment overlaps with SP_7–11_ (Phe-Phe-Gly-Leu-Met-NH_2_).	Coactivates MOR and substance-P/NK1 receptors. Produces opioid-dependent spinal analgesia without tolerance development in repeated administration paradigms; NK1/SP receptor blockade restores tolerance.	[73]
**Wtorek compounds 4**	Cyclic opioid scaffold Tyr-[D-Lys-Phe-Phe-Asp] linked to an SP6–11-like fragment: Gln-Phe-Phe-Gly-Leu-Met-NH2. Full sequence: Tyr-[D-Lys-Phe-Phe-Asp]-Gln-Phe-Phe-Gly-Leu-Met-NH2.	MOR/KOR agonist and NK1 agonist. Reported pEC_50_ values: MOR = 7.46 nM, KOR = 7.85 nM, NK1 = 8.45. Produced dose-dependent antinociception without tolerance or constipation in mice.	[12]
**Wtorek compounds 5–7**	Cyclic opioid scaffold Tyr-[D-Lys-Phe-Phe-Asp] linked to shortened SP fragments: compound 5 = SP_7–11_ (Phe-Phe-Gly-Leu-Met-NH2); compound 6 = SP_8–11_ (Phe-Gly-Leu-Met-NH2); compound 7 = SP_9–11_ (Gly-Leu-Met-NH2).	MOR/KOR agonists. NK1 agonist potency decreased with truncation of the SP fragment (pEC50: 5 = 7.80; 6 = 6.11; 7 = 5.68). These analogs were not tested in in vivo tolerance/constipation studies.	[12]
**Wtorek compounds 2 and 3**	Cyclic opioid scaffold Tyr-[D-Lys-Phe-Phe-Asp] linked to Spantide II C-terminal antagonist fragments. Compound 2: Asn-D-Trp-Phe-D-Trp-Leu-Nle-NH2; compound 3: D-Trp-Phe-D-Trp-Leu-Nle-NH_2_.	MOR/KOR agonist and NK1 antagonist profile. NK1 Ki and pKB values: for peptide 2 = 10.48 nM and 12.82 nM; for peptide 3 = 7.33 nM and 7.63 nM. Compound 2 produced antinociception without tolerance or constipation in mice.	[12]
**AA3052** **DALDA/substance P derivatives**	Tyr-D-Arg-Phe-Lys-D-Phe-Phe-D-Phe-Leu-Leu-NH_2_; DALDA is a MOR-selective dermorphin-related opioid peptide combined with substance P analog, D-Phe-Phe-D-Phe-Leu-Leu-NH_2_.	MOR agonist/NK1 agonist chimera. Produced antinociceptive effects against mechanical and thermal noxious stimuli after i.c.v. administration and resisted tolerance development.	[76]
**AWL3106**	Dermorphin/SP_7–11_ agonist–agonist chimera Tyr-D-Ala-Phe-Gly-Tyr-Pro-Ser-D-Ala-Phe-Phe-Gly-Leu-Met-NH_2_, with a spacer that enables the two pharmacophores to act independently.	Opioid/tachykinin agonist hybrid with high analgesic potency and markedly reduced ability to induce tolerance and dependence; also evaluated for cardiorespiratory actions in rats.	[77]
**AA501**	Compact opioid/tachykinin antagonist chimera based on Tyr-D-Ala-Gly-Phe linked through hydrazide/carboxybenzyl-Trp antitachykinin moiety; closely related to AA3266, differing in Trp stereochemistry.	Opioid receptor agonist/tachykinin receptor antagonist. Produced spinal antinociception in acute and neuropathic pain models after intrathecal administration; repeated administration led to tolerance, accelerated by coadministration of substance P.	[78]
**AA3266**	AA501 stereochemical analog: Tyr-D-Ala-Gly-Phe-NH-NH-Z-D-Trp. Combines an opioid agonist pharmacophore with an NK1R antagonist carboxybenzyl-D-Trp/hydrazide moiety.	Opioid agonist/NK1R antagonist. Reported high affinity for MOR and DOR and moderate NK1R binding; potent antinociception comparable to morphine, less tolerance than morphine and no constipation; also, selective cytotoxicity toward several cancer cell lines.	[79]
**AWL3020**	Hybrid of a [D-Ala2, Leu6]-deltorphin-derived DOR agonist segment (Tyr-D-Ala-Phe-His-Leu-Leu-Asp...) and a Spantide I-like NK1R antagonist segment (...D-Phe-Phe-D-Phe-Leu-Leu-NH_2_).	DOR agonist/NK1R antagonist design. High DOR affinity (IC_50_ = 29.5 nM), but poor NK1R affinity (IC_50_ = 70.28 uM). Analgesic action was weaker than morphine; cytotoxicity occurred only at higher concentrations and was not selective.	[80]
**TY027/C-terminal-modified opioid-NK1 antagonist lead peptide**	Tyr-D-Ala-Gly-Phe-Met-Pro-Leu-Trp-NH-[3′,5′-(CF3)2Bzl]. Prototype C-terminal-modified peptide combining an opioid address/message sequence with a 3,5-bis(trifluoromethyl)benzyl NK1 antagonist pharmacophore.	MOR/DOR agonist and NK1R antagonist profile. Served as a lead scaffold for later TY005, glycosylated, Dmt-substituted, cystine-bridged and penicillamine-constrained bifunctional analgesic peptides.	[69]
**TY005**	Tyr-D-Ala-Gly-Phe-Met-Pro-Leu-Trp-O-3,5-Bn(CF3)2. Peptidic opioid agonist/NK1 antagonist derived from the TY027 family, with an ester-linked C-terminal 3,5-bis(trifluoromethyl)benzyl antagonist element.	Produced dose-dependent antinociception in non-injured rats and reversed nerve injury-induced tactile and thermal hypersensitivity more effectively than morphine in the SNL model. No rotarod-detectable motor impairment was observed. Repeated spinal administration did not produce antihyperalgesic tolerance.	[70]
**NP30/truncated TY005 derivative**	Tyr-D-Ala-Gly-Phe-Gly-Trp-O-[3′,5′-Bzl(CF3)2]. Six-residue truncated TY005-derived peptide in which the central Met-Pro-Leu region was shortened while retaining opioid and NK1 antagonist pharmacophoric elements.	Maintained balanced opioid agonist and substance-P/NK1 antagonist activities in isolated tissue assays. Reported to show significant antinociceptive and antiallodynic effects in vivo despite sequence truncation.	[81]
**O-beta-glycosylated TY027 derivatives**	TY027 analogs incorporating O-beta-glucosylated serine [Ser(Glc)] within the peptide sequence to increase metabolic stability and alter pharmacokinetic behavior while preserving opioid/NK1 dual pharmacology.	Glycosylation improved metabolic stability; analogs with glycosylation close to the Trp/NK1 antagonist region showed the most favorable balance of opioid agonism and NK1 antagonism in the reported series. Developed to improve the short half-life of linear peptide hybrids.	[82]
**TY038/cystine-bridged TY027 analog**	Tyr-cyclo[D-Cys-Gly-Phe-Met-Pro-D-Cys]-Trp-NH-[3′,5′-(CF3)2Bzl]. Cystine-constrained TY027 analog designed to stabilize the peptide backbone and improve plasma stability.	Highly DOR-selective opioid agonist and NK1 antagonist with good metabolic stability; reported plasma half-life exceeded 24 h in rat plasma.	[83]
**c[D-Pen2,Pen5]TY027/penicillamine-constrained analog**	Tyr-cyclo[D-Pen-Gly-Phe-Pen]-Pro-Leu-Trp-NH-[3′,5′-(CF3)2Bzl]. Topologically constrained TY027 analogue incorporating two penicillamine residues.	Potent opioid agonist and effective NK1 antagonist; approximately 1400-fold DOR selectivity over MOR. The compound was highlighted as metabolically stable and a promising research tool for analgesic design.	[71]
**Dmt1-TY027 analogs**	TY027-related peptides in which the N-terminal Tyr was replaced by 2′,6′-dimethyl-L-tyrosine (Dmt), a modification introduced to enhance opioid affinity, efficacy and metabolic stability.	Dmt substitution improved biological activity within the MOR/DOR opioid agonist–NK1 antagonist series. Reported as potent and efficacious peptide derivatives with antinociceptive activity and no detectable motor impairment or opioid tolerance in preclinical evaluation.	[84]
**SBCHM01/compact opioid–NK1 chimera**	Compact peptide-derived chimera based on the MOR-active tetrapeptide H-Dmt-D-Arg-Aba-Gly-NH_2_ (AN81) coupled at the C-terminus to a 3′,5′-bistrifluoromethylbenzyl NK1 antagonist moiety.	Dual opioid agonist/NK1 antagonist in vitro profile. Produced significant systemic antinociception for approximately 2–3 h, but did not prevent opioid-induced tolerance in the study; useful comparator showing that compactness alone does not guarantee improved chronic opioid-like profile.	[85]

**Table 3:** Hybrid peptides targeting opioid–neurotensin receptors.

Compound/ Family	Structure or Design	Main Profile	Key Reference
**PK20**	H-Dmt-D-Lys-Phe-Phe-Lys-Lys-Pro-Phe-Tle-Leu-OH. A decapeptide opioid–neurotensin chimera: modified endomorphin-2 opioid pharmacophore fused/overlapped with a modified NT(8–13)-like C-terminal fragment. Main substitutions include Tyr1 → Dmt, Pro2 → D-Lys, Arg8/Arg9 → Lys/Lys and Ile12 → Tle.	Strong central and peripheral antinociceptive activity in tail-flick test. The effect was only partially reversed by naltrexone, supporting involvement of both opioid and non-opioid/neurotensin components. Reported to cross the blood–brain barrier after peripheral administration.	[101]
**[Ile9]PK20**	H-Dmt-D-Lys-Phe-Phe-Lys-Lys-Pro-Phe-Ile-Leu-OH. Structural analog of PK20 in which the tert-leucine residue of PK20 is replaced by isoleucine. Maintains the opioid–neurotensin hybrid pharmacophore arrangement.	Active opioid–neurotensin hybrid analgesic, but with lower potency/efficacy than PK20 in reported comparisons. Chemical-stability studies showed that replacing Tle with Ile did not improve the overall stability profile; PK20 was generally more stable under several stress conditions.	[102]
**PK23**	Tyr-D-Ala-Phe-Phe-Lys-Lys-Pro-Phe-Ile-Leu-OH. Third member of the PK opioid–neurotensin series. Compared with PK20/[Ile9]PK20, PK23 lacks Dmt and Tle but includes D-Ala at position 2 and retains a modified NT-like C-terminal fragment.	Spinally active hybrid peptide with long-lasting antinociception. Intrathecal administration produced potent pain relief, delayed tolerance development compared with morphine, reduced scratch reflex and no major motor impairment at effective spinal doses. Antinociception was attenuated by opioid and NTS1 antagonism, while NTS2 antagonism did not significantly alter the response in the reported experiment.	[99]
**KGOP01/NT(8–13) opioid–neurotensin hybrids; peptide 7**	Design platform based on fusion of the metabolically stable MOR/DOR agonist tetrapeptide KGOP01, H-Dmt-D-Arg-Aba-β-Ala-NH_2_, with modified NT(8–13) analogs. Peptide 7 incorporates Dmt and β^3^-homoarginine within the NT-derived portion to improve NTS2 affinity/selectivity.	MOR agonist with extremely high NTS2 affinity reported in the picomolar range (K_i_ about 3 pM) and good NTS1 affinity (K_i_ about 4 nM), giving >1300-fold NTS2 selectivity. In mice, peptide 7 produced significant and prolonged antinociception lasting up to 8 h, compared with the parent KGOP01.	[103]
**KGOP01/NT(8–13) analog 9**	Analog from the KGOP01-fused opioid–neurotensin series containing a (6-OH)Tic substitution in the NT-derived part. Designed to reduce NTS1 binding while preserving NTS2 affinity.	High NTS2 affinity (reported K_i_ about 1.7 nM) with much lower NTS1 affinity (reported K_i_ about 4.7 micromolar), giving strong NTS2 selectivity (>2700). Useful SAR example showing how Tyr11/(6-OH)Tic-type substitutions can shift the NTS1/NTS2 profile.	[103]
**SBL-OPNT-05 and SBL-OPNT-10**	General design: the MOR/DOR agonist H-Dmt-D-Arg-Aba-β-Ala-NH_2_ linked to NT(8–13) analogs optimized for NTS2 affinity. The research focused on linker length, naphthyl/Aba-related changes, beta3hArg at Arg5 and modifications in the NT pharmacophore.	Lead/template compounds for a new generation of proteolytically stable opioid–neurotensin hybrid peptidomimetics. The series was developed to retain MOR/DOR activity while improving NTS2 selectivity and metabolic stability, with reduced β-arrestin 2 recruitment at MOR as a desirable profile.	[104]
**SBL-OPNT-13**	H-Dmt-D-Arg-Aba-Gly-Arg-Arg-Pro-Tic(6-OH)-Tle-Leu-OH. OPNT peptidomimetic combining a modified opioid tetrapeptide pharmacophore with an NTS2-optimized neurotensin-like fragment containing Tic(6-OH) and Tle.	One of the most promising OPNT analogs. Reported excellent NTS2 selectivity (NTS2 K_i_ about 2.68 nM), meaningful MOR/DOR affinity, low β-arrestin 2 recruitment efficacy at MOR, partial G_αi1_ agonism with improved potency *vs* DAMGO, and rat-plasma half-life > 24 h. Produced antinociceptive effects in acute tail-flick, postoperative pain and formalin models.	[104]
**SBL-OPNT-14**	H-Dmt-D-Arg-Aba-GABA-Arg-Arg-Pro-Tic(6-OH)-Tle-Leu-OH. Close analog of SBL-OPNT-13 in which the linker is GABA rather than Gly.	High DOP affinity (reported K_i_ about 12 nM). Useful SAR example showing how linker length/composition can alter the balance between opioid receptor binding and neurotensin receptor selectivity.	[104]
**SBL-OPNT-16 and SBL-OPNT-17**	SBL-OPNT-16: H-Dmt-D-Arg-1Ana-Gly-Arg-Arg-Pro-Tic(6-OH)-Tle-Leu-OH. SBL-OPNT-17: H-Dmt-D-Arg-1Ana-GABA-Arg-Arg-Pro-Tic(6-OH)-Tle-Leu-OH. Both include 1Ana in the opioid pharmacophore and Tic(6-OH)/Tle in the NT-like fragment.	Highly NTS2-selective analogs. Reported NTS2 K_i_ values were about 3.01 nM for SBL-OPNT-16 and 1.03 nM for SBL-OPNT-17. These compounds illustrate the effect of replacing Aba with naphthyl-type residues and modulating the linker while preserving a strong NTS2 profile.	[104]
**SBL-OPNT-18**	H-Dmt-D-Arg-Aba-Gly-beta3hArg-Arg-Pro-Tic(6-OH)-Tle-Leu-OH. Analog of the SBL-OPNT series incorporating beta3hArg in the NT-derived region.	Among the analogs highlighted for reduced β-arrestin 2 recruitment efficacy at MOP (about 12% in the reported assay) and partial G_αi1_ agonism. Rat-plasma half-life exceeded 24 h in the study, supporting the peptidomimetic stability rationale.	[104]
**BNT12**	Novel hybrid peptide containing opioid and neurotensin-like fragments: Tyr-D-Ala-Gly-Phe-Gly-Gly-Arg-Arg-Pro-Tyr-Ile-Leu-OH.	Potent central antinociception after i.c.v. administration in mice. Activity was reported to involve MOR and DOR as well as NTSR1 and NTSR2. BNT12 showed effects in acute thermal, SNI neuropathic, CFA inflammatory, visceral and formalin pain models, with reduced acute tolerance, insignificant chronic tolerance at supraspinal level and reduced/no opioid-like side effects in several behavioral and physiological assays.	[105]

**Table 4:** Opioid/neuropeptide FF hybrid peptides and peptide-derived ligands.

Compound/ Family	Structure or Design	Main Profile	Key Reference
**NPYFa–Met-enkephalin–NPFF chimera**	Chimeric peptide reported as Tyr-Gly-Gly-Phe-Met-Lys-Lys-Lys-Pro-Gln-Arg-Phe-NH_2_. Designed by combining a Met-enkephalin-like opioid motif with the Pro-Gln-Arg-Phe-amide (PQRFamide) C-terminal motif related to NPFF.	Multireceptor opioid/NPFF-related ligand. Produced early-onset, dose-dependent and prolonged antinociception; antagonist experiments suggested a predominant KOR-mediated antinociceptive component. Eu-GTP-gamma-S assays indicated interaction with opioid and NPFF receptors. Chronic 6-day treatment did not produce analgesic tolerance in rodents.	[132]
**EN-9–endomorphin-2–NPFF chimera**	Tyr-Pro-Phe-Phe-Gln-Pro-Gln-Arg-Phe-NH_2_. The N-terminal Tyr-Pro-Phe-Phe sequence derives from endomorphin-2, whereas the C-terminal Gln-Pro-Gln-Arg-Phe-NH_2_ region corresponds to an NPFF-like RFamide pharmacophore.	Multifunctional opioid/NPFF receptor agonist profile, described especially as a KOR, NPFFR1 and NPFFR2 agonist. Produced potent antinociception after central and systemic administration with limited tolerance, rewarding effect and constipation-like liability in preclinical models.	[127,133]
**BN-9–biphalin–NPFF chimera**	Tyr-D-Ala-Gly-Phe-Gln-Pro-Gln-Arg-Phe-NH_2_ (YdAGFQPQRF-NH_2_). Designed by fusing an opioid pharmacophore related to the N-terminal half-structure of biphalin with the NPFF(3–8)-derived Gln-Pro-Gln-Arg-Phe-NH_2_ fragment.	Mixed opioid/NPFF receptor agonist. In cAMP assays, BN-9 acted at MOR, DOR, KOR, NPFFR1 and NPFFR2. In the tail-flick assay it produced strong dose-dependent antinociception approximately equipotent to morphine; it was effective also under inflammatory conditions. Antinociception was mainly mediated by MOR and KOR. Repeated administration produced robust antinociception without tolerance and with limited inhibition of gastrointestinal transit.	[128]
**DN-9/optimized BN-9 derivative**	Tyr-D-Ala-Gly-N-Me-Phe-Gly-Pro-Gln-Arg-Phe-NH_2_. Structure-based optimization of BN-9; replacement within the overlapping opioid/NPFF pharmacophore introduced N-methylphenylalanine and Gly to improve potency and pharmacological profile.	Multifunctional opioid/NPFF receptor agonist with potent nontolerance-forming antinociception. Reported as markedly more potent than BN-9 in acute assays. Analgesia is mediated mainly by MOR and KOR and the compound retained activity in inflammatory and neuropathic pain models. Subcutaneous DN-9 produced antinociception in inflammatory and neuropathic pain through peripheral MOR and KOR mechanisms. The efficacy was maintained over 8 days of repeated treatments. The compound retained antinociceptive activity in morphine-tolerant mice.	[134,135]
**Cyclic disulfide BN-9 analogs**	Cyclized analogs of BN-9 containing L- or D-cysteine residues at selected positions to form disulfide-constrained opioid/NPFF receptor agonists. Example design: cyclic Tyr-[Cys/D-Cys-Gly-Phe-Cys]-Pro-Gln-Arg-Phe-NH_2_-type scaffold.	Cyclic and corresponding linear analogs behaved as multifunctional agonists at opioid and NPFF receptors. Disulfide cyclization improved stability, prolonged duration of antinociception and preserved the nontolerance-forming profile relative to the parent BN-9 series.	[136]
**Orally active cyclic DN-9 analogs**	Side-chain cyclized DN-9 analogs designed to improve metabolic stability and restrict central exposure. Representative analog **1** reported as Tyr-c[D-Lys-Gly-N-Me-Phe-Asp]-Pro-Gln-Arg-Phe-NH_2_.	Multifunctional opioid/NPFF receptor agonists producing potent, long-lasting and mainly peripherally restricted antinociception after subcutaneous and oral administration. Orally administered analog **1** showed no significant tolerance, dependence, constipation or respiratory depression at effective analgesic doses.	[130]
**OFP011/cyclic DN-9 analog**	Tyr-c^2,5^[D-Cys-Gly-N-Me-Phe-Cys]-Pro-Gln-Arg-Phe-NH_2_. Cyclic disulfide analog of DN-9, commonly described as c[D-Cys2,Cys5]-DN-9. Designed to improve metabolic stability, systemic efficacy and blood–brain barrier penetration relative to the parent linear multifunctional peptide.	Multifunctional opioid/NPFF receptor agonist. In vitro, OFP011 showed agonistic activity at MOR and NPFFR1/NPFFR2 and partial agonism at DOR/KOR. Systemic and oral administration produced potent and long-lasting antinociception in acute, neuropathic and inflammatory pain models, with limited tolerance, reward, motor impairment, respiratory depression and gastrointestinal inhibition in the tested dose range.	[137]
**EKR–branched endomorphin-2–RF9 peptidomimetic**	Branched peptidomimetic combining endomorphin-2 and the NPFF receptor antagonist RF9 pharmacophores. Reported sequence/design: Tyr-Pro-Phe-Phe-Lys(NH_2_)-Phe-Arg-1-Aca.	Mixed opioid/NPFF receptor ligand. MOR and DOR agonism combined with NPFFR1 antagonism and NPFFR2 partial agonism. Produced centrally mediated antinociception in the tail-flick test.	[138]
**RKE–reverse branched EM-2–RF9 peptidomimetic**	Reverse-orientation branched analog of EKR, also combining EM-2 and RF9 pharmacophores. Reported sequence/design: 1-Aca-Arg-Phe-Lys(NH_2_)-Phe-Phe-Pro-Tyr.	MOR agonist combined with NPFFR1 antagonist/NPFFR2 partial agonist activity. Produced central antinociception, but with a receptor profile distinct from EKR, illustrating that pharmacophore orientation affects opioid/NPFF balance.	[138]
**KGFF03–biased MOR agonist-NPFFR agonist**	H-Dmt-D-Arg-Aba-β-Ala-Arg-Phe-NH_2_. Peptidomimetic derived from a compact MOR-active opioid pharmacophore and an NPFF-related C-terminal Arg-Phe-amide motif.	G protein-biased MOR agonist with NPFFR1/NPFFR2 agonist activity. Served as a comparator showing that MOR bias can reduce some acute MOR-related liabilities, whereas NPFF receptor agonism does not necessarily provide the most favorable chronic opioid-like profile, compared with NPFFR antagonism.	[125]
**KGFF09–biased MOR agonist-NPFFR antagonist**	H-Dmt-D-Arg-Aba-β-Ala-Bpa-Phe-NH_2_. Peptidomimetic combining a G protein-biased MOR agonist pharmacophore with an NPFFR antagonist pharmacophore.	Bifunctional MOR agonist/NPFFR antagonist. Reported to produce antinociception in acute, persistent inflammatory and visceral pain models after systemic administration. Compared with classical opioids, it showed reduced propensity for respiratory depression, reward-like effects, tolerance, OIH and withdrawal-like responses in preclinical studies.	[139]
**DP08/DP14/DP32/DP50 KGFF09-optimized series**	Optimized multitarget peptide/peptidomimetic analogs derived from the KGFF09 design principle. DP08/DP32 structures: Dmt-N-Me-D-Ala-1-Ana-Gly/β-Ala or Dmt/guanidyl-Dmt-D-Pro/D-Arg-Aba- β-Ala opioid pharmacophores linked to Bpa-Phe-type NPFFR antagonist pharmacophore; DP32 and DP50 were selected as improved in vivo leads.	MOR agonist/NPFFR antagonist series. DP32 and DP50 showed potent antinociception that was mostly peripherally mediated and, compared with morphine and TRV130, produced no respiratory depression, no hyperalgesia, and significantly less tolerance and strongly attenuated withdrawal syndrome in mice.	[126]
**MCRT/morphiceptin–PFR(Tic)-amide chimera**	Tyr-Pro-Phe-Pro-Phe-Arg-Tic-NH_2_, chimeric peptide based on the MOR-preferring morphiceptin motif Tyr-Pro-Phe-Pro-NH_2_ and the NPFF-related Pro-Phe-Arg-Tic-NH_2_ pharmacophore sharing one Pro residue.	Multifunctional opioid/NPFF-related ligand. Produced dose- and time-dependent antinociception in the mouse tail-flick test after i.c.v. administration; activity involved mixed MOR/DOR mechanisms and partial NPFF receptor interaction. Attenuated neuropathic pain in the spared nerve injury model.	[140,141]

**Table 5:** Opioid agonist/CCK2R antagonist hybrid peptides and peptide-derived ligands.

Compound/ Family	Structure or Design	Main Profile	Key Reference
**CCK octapeptide analgesic analogs/early opioid-CCK overlap leads**	Modified CCK octapeptide analogs with high affinity for CCK2R; studied as analgesic CCK analogs with additional opioid receptor binding, especially at DOR.	Early evidence that some CCK2R-selective peptide analogs with analgesic activity after i.c.v. administration also display opioid receptor binding properties. These compounds should be treated as pharmacophore-overlap precursors rather than fully optimized opioid agonist/CCK antagonist hybrids.	[157]
**SNF9007/historical CCK-opioid pharmacophore-overlap lead**	Asp-Tyr-D-Phe-Gly-Trp-(N-Me)Nle-Asp-Phe-NH_2_. CCK(26–33)-derived analog designed for CCK2R recognition and later shown to interact functionally with opioid receptors.	Produced antinociception after i.c.v. administration in mouse hot-plate and warm-water tail-flick tests. CCK1R or CCK2R antagonists did not block the effect, whereas combined MOR + DOR antagonism strongly reduced antinociception. It should be classified as a historical CCK/opioid pharmacophore-overlap lead, not as a CCK antagonist hybrid.	[158]
**Hruby/Agnes opioid agonist-CCK antagonist prototype peptides**	Peptide ligands derived from the concept that CCK-2 and opioid receptors share overlapping pharmacophoric/topographical requirements. Designed to combine MOR/DOR opioid receptor agonism with CCK receptor antagonism in a single molecule.	This entry represents a design concept/prototype family rather than one fully developed in vivo lead compound. Especially, compound **6** (Tyr-D-Phe-Gly-D-Trp-N-Me-Nle-Asp-Phe-NH_2_) showed high functional activity towards opioid and CCK2 receptors.	[159]
**Agnes compound 9/overlapping opioid-CCK pharmacophore peptide**	Tyr-D-Nle-Gly-Trp-Nle-Asp-Phe-NH_2_. Linear peptide designed by overlapping structural elements required for opioid and CCK receptor recognition.	Displayed potent agonist activity at MOR/DOR in isolated tissue assays and balanced binding to CCK1R and CCK2R.	[8]
**Agnes compound 11/CCK2-preferring opioid-CCK peptide**	Tyr-D-Ala-Gly-D-Trp-N-MeNle-Asp-Phe-NH_2_. Linear overlapping-pharmacophore peptide containing D-Trp and N-methyl-norleucine modifications.	Reported opioid agonist properties at MOR/DOR and high CCK2R binding affinity relative to CCK1R. The compound illustrates how D-amino acid and N-methylated residues tune opioid/CCK receptor balance and CCK receptor subtype preference.	[8]
**Hydrazide-linked opioid agonist/CCK antagonist peptides**	General design: opioid tetrapeptide pharmacophore Tyr-D-Ala-Gly-Phe- linked through a hydrazide spacer to CCK-derived antagonist fragments, for example, Tyr-D-Ala-Gly-Phe-NH-NH-Phe-Asp-Nle-D-Trp-Boc and related Boc/Adoc/Ac/N-MeNle analogs.	Designed as MOR/DOR opioid receptor agonists and CCK1R/CCK2R antagonists. Many analogs retained high opioid receptor affinity; CCK receptor binding was often weaker, but several compounds behaved as potent CCK receptor antagonists in isolated tissue assays. These are among the clearest peptide-derived opioid agonist/CCK antagonist hybrid families.	[160]
**Partial retro-inverso, retro and inverso hydrazide-linked analogs**	Modified isomers of hydrazide-linked opioid/CCK bifunctional peptides in which the CCK pharmacophore was reorganized by partial retro-inverso, retro or inverso modifications.	Used to probe pharmacophore topology and improve receptor balance. Modifying the CCK pharmacophore changed CCK1R/CCK2R and opioid bioactivities, with reported increases in CCK binding affinity and altered selectivity in selected analogs. Can serve as potential tools for SAR studies.	[161]
**Cyclic disulfide opioid-CCK peptides**	Conformationally constrained cyclic disulfide analogs based on overlapping opioid/CCK pharmacophores, for example, Tyr-c[D-Cys-Gly-Trp-Cys]-Asp-Phe-NH_2_ and related analogs.	Cyclization was introduced to constrain the peptide backbone and define three-dimensional requirements for opioid and CCK receptor recognition. Some analogs showed potent MOR/DOR agonist properties; CCK receptor activity varied and was generally less optimized than opioid activity. A series that can be useful in further SAR series.	[162]
**Bivalent MOP agonist/CCK2 antagonist ligands**	Bivalent ligands containing MOR agonist pharmacophore and a CCK2R antagonist pharmacophore separated by variable-length linkers. Examples include compounds 4a-4c with 9-, 16- and 22-atom spacers.	Longer spacers promoted MOR-CCK2R receptor association in BRET-based cellular assays, whereas the 9-atom spacer did not. These are receptor-crosstalk probes rather than classical peptide–peptide hybrids.	[163]
**Trivalent opioid/CCK/melanocortin ligands**	Peptide-derived multitarget constructs combining opioid, CCK and melanocortin pharmacophores in one molecule. Ligand **10** (Dmt-D-Ala-Gly-Phe-His-2′-D-Nal-Trp-Nle-Asp-Phe-NH2) was reported as the lead compound with the best activity balance at all three target systems.	Extends the opioid/CCK hybrid concept by adding melanocortin receptor antagonism, another anti-opioid/pronociceptive system implicated in chronic pain and opioid tolerance.	[164]

**Table 6:** Opioid/melanocortin hybrid peptides, peptidomimetics and peptide-derived multitarget ligands.

Compound/ Family	Structure or Design	Main Profile	Key Reference
**Opioid/melanocortin overlapping-pharmacophore peptidomimetics**	Peptidomimetic series built by overlapping Dmt-Tic DOR pharmacophore with a melanocortin pharmacophore related to Tic-D-Phe(*p*-Cl)-piperidin-4-yl-N-phenylpropionamide. Included as an early SAR/proof-of-concept, not as a classical peptide-peptide hybrid or an in vivo MC4 antagonist analgesic lead.	Compound **1** displayed high DOR agonist activity (K_i_ = 0.38 nM in binding, EC_50_ = 0.48 nM in GTP-gamma-S, IC_50_ = 74 nM in MVD) and selective MC3R receptor binding (IC_50_ = 2.3 micromolar). This supports the concept of overlapping opioid and melanocortin pharmacophores through Tic, but does not establish an opioid/MC4 bifunctional analgesic profile in vivo.	[185]
**Early Kulkarni/Hruby opioid-MC4R bifunctional ligand analogs**	Early peptide/peptidomimetic analogs designed to combine opioid receptor agonist elements with an MC4R antagonist pharmacophore. Included as a conference/proceedings-level conceptual precursor rather than a detailed in vivo pharmacology source.	Useful design precedent for opioid-MC4R multitarget analgesic development. The reference confirms the existence of the early analog series, but broad claims about analgesic efficacy, tolerance, constipation, respiratory depression or dependence should not be based on this short proceedings paper alone.	[186]
**Trivalent opioid/CCK/melanocortin ligands**	Trivalent peptide-derived ligands combining opioid, cholecystokinin and melanocortin pharmacophores in one scaffold. Included only as an expanded multitarget design example; not a pure opioid/melanocortin hybrid and not a simple bifunctional opioid-MC4R analgesic lead.	Designed to conserve topographical pharmacophore structures for all three receptor systems. The study is useful as a synthetic/design precedent for multitarget pain ligands, but the entry should not be used to support claims specific to pure opioid/MC4R bifunctional pharmacology.	[164]
**UW3/ENK-Ahx-SHU/OP-linker-MC4 active lead**	Tyr-D-Ala-Gly-Phe-Ahx-Nle-cyclo[Asp-His-D-Nal(2′)-Arg-Trp-Lys]-NH_2_. Tyr-D-Ala-Gly-Phe linked through Ahx [approximately -NH-(CH2)5-CO-] to a SHU9119-derived melanocortin antagonist fragment: Nle-cyclo[Asp-His-D-Nal(2′)-Arg-Trp-Lys]-NH_2_. The melanocortin fragment is MC4R-targeting in the cited pain studies but derives from SHU9119, which is not absolutely MC4R-selective.	Well-supported opioid agonist/MC4R-targeting antagonist peptidomimetic. In CCI mice after i.t. administration, ENK-Ahx-SHU showed strong activity in neuropathic-pain assays: ED_50_ = 0.0002 nM in the von Frey test and 0.004 nM in the cold-plate test. Binding data for ENK-Ahx-SHU: MOR IC_50_ = 64.03 ± 34.64 nM, DOR IC50 = 45.43 ± 32.33 nM and MC4R IC_50_ = 1.83 ± 0.81 nM. A CCI study in rats further supports potent and long-lasting antihypersensitivity effects and delayed tolerance relative to morphine after repeated i.t. dosing.	[183]
**UW5/ENK-(Ahx)2-SHU/long flexible linker hybrid**	Tyr-D-Ala-Gly-Phe-Ahx-Ahx-Nle-cyclo[Asp-His-D-Nal(2′)-Arg-Trp-Lys]-NH_2_. Tyr-D-Ala-Gly-Phe connected to the SHU9119-derived melanocortin antagonist fragment through two Ahx units: -NH-(CH2)5-CO-NH-(CH2)5-CO-.	Opioid agonist/MC4R antagonist with potent analgesic activity in neuropathic pain model. In the SAR study, ENK-(Ahx)2-SHU showed MOR IC_50_ = 5.47 ± 1.38 nM, DOR IC_50_ = 22.32 ± 12.67 nM and MC4R IC_50_ = 0.50 ± 0.16 nM. In a CCI experiment in mice after i.t. administration, ED_50_ values were 0.01 nM in the von Frey test and 0.1 nM in the cold-plate test. Rat CCI studies support potent antinociceptive/antihypersensitivity effects and delayed tolerance compared with morphine.	[184]
**[Dmt1]ENK-Ahx-SHU/compound 9**	Dmt-D-Ala-Gly-Phe-Ahx-Nle-cyclo[Asp-His-D-Nal(2′)-Arg-Trp-Lys]-NH_2_. Dmt replaces Tyr in the opioid pharmacophore, and Ahx connects the opioid and melanocortin antagonist fragments.	Dmt-substituted opioid/melanocortin peptidomimetic. In CCI-exposed mice after i.t. administration, compound **9** showed ED_50_ = 0.003 nM in von Frey and 0.009 nM in cold-plate testing. It had weak or non-calculable acute tail-flick activity, supporting relative preference for neuropathic-pain assays rather than acute nociception under the reported conditions.	[9]
**ENK-D-Ala-SHU and ENK-β-Ala-SHU/short flexible linker analogs**	Tyr-D-Ala-Gly-Phe-X-Nle-cyclo[Asp-His-D-Nal(2′)-Arg-Trp-Lys]-NH_2_, where X is D-Ala or β-Ala. These are short flexible-linker analogs of the ENK-SHU series.	SAR comparators rather than best leads. The short-linker analogs showed strong MC4R binding (MC4R IC_50_ = 0.07 ± 0.01 nM and 0.12 ± 0.01 nM, respectively). In vivo profiles were less favorable than Ahx-containing analogs: compound **1** showed weak/poorly dose-dependent effects, whereas compound **2** was less potent than ENK-Ahx-SHU in the neuropathic model and in the cold-plate test.	[9]
**ENK-4AMB-SHU and ENK-4APhAc-SHU/rigid aromatic linker analogs**	Opioid/melanocortin peptidomimetics containing ENK and SHU9119-derived fragments connected by rigid aromatic linkers: 4-aminomethylbenzoic acid (4AMB) or 4-aminophenylacetic acid (4APhAc).	SAR analogs testing whether rigid aromatic linkers maintain dual receptor recognition and antinociception. In the 2022 CCI mouse study, ENK-4AMB-SHU and ENK-4APhAc-SHU showed antinociceptive activity in neuropathic-pain assays. ENK-4APhAc-SHU also displayed calculable acute tail-flick activity, so it should not be described as strictly neuropathic-pain selective.	[9]
**ENK-Pro-Gly-SHU and ENK-(Pro-Gly)2-SHU/semirigid proline-containing linker analogs**	Opioid/melanocortin hybrids containing the ENK opioid pharmacophore and SHU9119-derived melanocortin antagonist pharmacophore connected by -Pro-Gly- or -(Pro-Gly)2- semirigid linkers.	SAR analogs used to test semirigid proline-containing spacers. ENK-Pro-Gly-SHU showed measurable but weaker/moderate neuropathic antinociception, whereas ENK-(Pro-Gly)2-SHU lacked measurable analgesic effect in the CCI von Frey and cold-plate tests, despite having calculable acute tail-flick activity.	[9]
**US11041010B2/generalized opioid-MC4R peptidomimetic patent family**	General formula A1-A2-A3-A4-X-Nle-cyclo[Asp-His-D-Nal(2′)-Arg-Trp-Lys]-NH_2_. A1 may include Tyr, Dmt or N-Me-Tyr; A2 may include D-Ala, D-Thr, D-Ser, D-Leu, D-Arg, D-Lys or D-Pro; A3 may be absent or Gly/Phe; A4 may be Phe/Trp; X comprises flexible or double flexible linkers. Patent claims should not be treated as equivalent to fully peer-reviewed pharmacological characterization.	Patent family defining a broader chemical space around opioid agonist/MC4R antagonist peptidomimetics for neuropathic pain. The patent reports UW3 and UW5 ED_50_ values in micrograms for mouse CCI models and claims use in neuropathic pain, but patent data should be separated from peer-reviewed in vivo efficacy and safety claims.	[187]

## Data Availability

No new data were created or analyzed in this study. Data sharing is not applicable to this article.

## Abbreviations

The following abbreviations are used in this manuscript:

Aba	4-amino-1,2,4,5-tetrahydro-2-benzazepin-3-one scaffold
AC	adenyl cyclase
ACTH	adrenocorticotropic hormone
Adoc	1-adamantyloxycarbonyl
Akt	protein kinase B
BBB	blood–brain barrier
beta3hArg	beta3-homoarginine
Bn	benzyl
Bpa	4-benzoyl-L-phenylalanine
BRET	bioluminescence resonance energy transfer
CaV2.2	voltage-gated calcium channel subtype 2.2 (N-type calcium channel)
cAMP	cyclic adenosine monophosphate
CCI	chronic constriction injury
CCK	cholecystokinin
CCK1R/CCK-A	cholecystokinin receptor type 1
CCK2R/CCK-B	cholecystokinin receptor type 2
CFA	complete Freund’s adjuvant
c-Fos	cellular Jun proto-oncogene protein
CGRP	calcitonin gene-related peptide
CHO-K1	Chinese hamster ovary K1 cells
CNS	central nervous system
CREB	cAMP response element-binding protein
DAG	diacylglycerol
DAMGO	[D-Ala^2^, *N*-MePhe^4^, Gly-ol]-enkephalin
D-Nal	D-naphthylalanine
Dmt	2,6-dimethyl-L-tyrosine
DOR	delta-opioid receptor
EC_50_	half-maximal effective concentration
ERK	extracellular signal-regulated kinase
GABA	gamma-aminobutyric acid
G_α_	G alpha subunit
G_α13_	G protein alpha subunit 13
G_αi1_	G protein alpha subunit i1
G_αoA_	G protein alpha subunit oA
G_αs_	G protein alpha subunit s
G_β_	G beta subunit
G_γ_	G gamma subunit
G_i/o_	inhibitory G protein family
GPI/LMMP	guinea pig ileum/longitudinal muscle-myenteric plexus
GPCR	G protein-coupled receptor
GRKs	G protein-coupled receptor kinases
GTPγS	guanosine 5′-O-[γ-thio]triphosphate
G_q/11_	G protein subfamily G_q/11_
HEK293	human embryonic kidney 293 cells
i.c.v.	intracerebroventricular
IC_50_	half-maximal inhibitory concentration
i.t.	intrathecal
IP3	inositol 1,4,5-trisphosphate
JNK	c-Jun N-terminal kinase
K_i_	inhibition constant
KOR	kappa-opioid receptor
MAPK	mitogen-activated protein kinase
MOR	mu-opioid receptor
MC1-5R	melanocortin 1-5 receptors
MVD	mouse vas deferens
N-MeNle	N-methyl-norleucine
Nav1.7	voltage-gated sodium channel NaV1.7
NF-κB	nuclear factor kappa B
NK1R	neurokinin-1 receptor
NK2R	neurokinin-2 receptor
NK3R	neurokinin-3 receptor
Nle	norleucine
NMDA	N-methyl-D-aspartate
NOP	nociceptin/orphanin FQ peptide receptor
NPAF	neuropeptide AF
NPFF	neuropeptide FF
NPFFR1/NPFF1R	neuropeptide FF receptor 1
NPFFR2/NPFF2R	neuropeptide FF receptor 2
NPSF	neuropeptide SF
NPVF	neuropeptide VF
NT	neurotensin
NTS1/NTSR1	neurotensin receptor type 1
NTS2/NTSR2	neurotensin receptor type 2
OIH	opioid-induced hyperalgesia
p38 MAPK	p38 mitogen-activated protein kinase
pEC_50_	negative logarithm of EC_50_
PI3K	phosphoinositide 3-kinase
PK	protein kinase
PKA	protein kinase A
PKC	protein kinase C
PLA2	phospholipase A2
PLC	phospholipase C
PLCβ	phospholipase C beta
POMC	proopiomelanocortin
PTX	pertussis toxin
RFRP-1	RFamide-related peptide-1
RFRP-3	RFamide-related peptide-3
RVM	rostral ventromedial medulla
SAR	structure–activity relationship
SH-SY5Y	human neuroblastoma SH-SY5Y cells
SNI	spared nerve injury
SP	substance P
Tic(6-OH)	6-hydroxy-1,2,3,4-tetrahydroisoquinoline-3-carboxylic acid
Tle	tert-leucine
